# Thiosemicarbazone Derivatives Developed to Overcome COTI-2 Resistance

**DOI:** 10.3390/cancers14184455

**Published:** 2022-09-14

**Authors:** Vivien Pósa, Alessia Stefanelli, Julia H. Bormio Nunes, Sonja Hager, Marlene Mathuber, Nóra V. May, Walter Berger, Bernhard K. Keppler, Christian R. Kowol, Éva A. Enyedy, Petra Heffeter

**Affiliations:** 1 Department of Inorganic and Analytical Chemistry, Interdisciplinary Excellence Centre and MTA-SZTE Lendület Functional Metal Complexes Research Group, University of Szeged, Dóm tér 7, H-6720 Szeged, Hungary; 2 Center for Cancer Research, Medical University of Vienna, Borschkegasse 8a, 1090 Vienna, Austria; 3 Institute of Inorganic Chemistry, Faculty of Chemistry, University of Vienna, Waehringer Strasse 42, 1090 Vienna, Austria; 4 Inorganic Chemistry Department, Institute of Chemistry, University of Campinas—UNICAMP, Campinas 13083-970, SP, Brazil; 5 Research Cluster ‘‘Translational Cancer Therapy Research’’, 1090 Vienna, Austria; 6 Centre for Structural Science, Research Centre for Natural Sciences, Magyar Tudósok Körútja 2, H-1117 Budapest, Hungary

**Keywords:** thiosemicarbazones, COTI-2, zinc, copper, iron, metal binding, stability constants, resistance, anticancer, p53

## Abstract

**Simple Summary:**

Besides zinc, also iron and copper need to be considered to play a role in the mode of action and resistance development of the anticancer thiosemicarbazone COTI-2 and its derivatives. One of these derivatives (COTI-NMe_2_) was discovered as an interesting new drug candidate with improved anticancer activity and resistance profile.

**Abstract:**

COTI-2 is currently being evaluated in a phase I clinical trial for the treatment of gynecological and other solid cancers. As a thiosemicarbazone, this compound contains an N,N,S-chelating moiety and is, therefore, expected to bind endogenous metal ions. However, besides zinc, the metal interaction properties of COTI-2 have not been investigated in detail so far. This is unexpected, as we have recently shown that COTI-2 forms stable ternary complexes with copper and glutathione, which renders this drug a substrate for the resistance efflux transporter ABCC1. Herein, the complex formation of COTI-2, two novel terminal N-disubstituted derivatives (COTI-NMe_2_ and COTI-NMeCy), and the non-substituted analogue (COTI-NH_2_) with iron, copper, and zinc ions was characterized in detail. Furthermore, their activities against drug-resistant cancer cells was investigated in comparison to COTI-2 and Triapine. These data revealed that, besides zinc, also iron and copper ions need to be considered to play a role in the mode of action and resistance development of these thiosemicarbazones. Moreover, we identified COTI-NMe_2_ as an interesting new drug candidate with improved anticancer activity and resistance profile.

## 1. Introduction

**Scheme 1:**
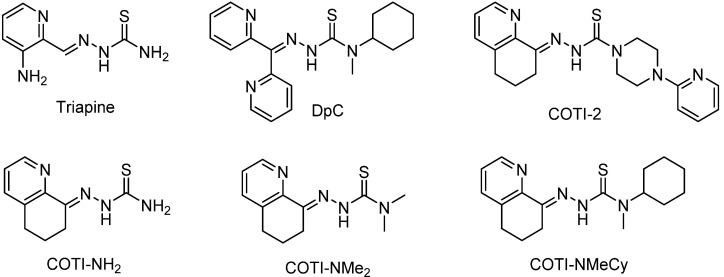
Chemical structures of the clinically investigated TSCs (Triapine, DpC, and COTI-2) as well as COTI-NH_2_ and the new derivatives, COTI-NMe_2_ and COTI-NMeCy.

α-*N*-Heterocyclic thiosemicarbazones (TSCs) are a prominent class of metal chelators that have been explored for the treatment of diverse human diseases, including cancer [1]. The mechanism of action of these drugs is closely related to complex formation with endogenous metal ions, such as iron and copper. The coordination of α-*N*-pyridyl TSCs takes place through an N,N,S-donor set resulting typically in mono-ligand complexes with copper(II) and bis-ligand species with iron(III/II) ions. Regarding cancer, TSCs were originally developed with the aim of targeting the enhanced demand for iron of the fast-dividing malignant cells [2]. Here, the iron-dependent enzyme ribonucleotide reductase (RR) was especially considered as the main target. Subsequently, the potent RR inhibitor 3-aminopyridine-2-carboxaldehyde thiosemicarbazone (Triapine, 3-AP) was tested in more than 30 clinical phase I and II trials alone or in combination with other anticancer drugs [3,4]. Notably, Triapine recently entered a clinical phase III trial in combination with cisplatin and radiation therapy for cervical or vaginal cancer patients (study number NCT02595879, www.clinicaltrials.gov (accessed on 20 August 2022)). However, just like in the case of other anticancer drugs, Triapine-based therapy has several drawbacks, such as a rather low plasma half-life as well as the occurrence of drug resistance [5,6]. Consequently, the evaluation of new derivatives is ongoing. Several α-*N*-heterocyclic TSCs have been developed with di-substituted terminal NH_2_ groups, as they display highly increased anticancer activity in the nanomolar concentration range in cell cultures [7,8]. Two lead candidates of this compound class, namely di-2-pyridylketone 4-cyclohexyl-4-methyl-3-thiosemicarbazone (DpC; Scheme 1) and 4-(pyridine-2-yl)-*N*-([(8E)-5,6,7,8-tetrahydroquinolin-8-ylidene]amino)piperazine-1-carbothioamide (COTI-2; Scheme 1), are currently being tested in phase I clinical trials (www.clinicaltrials.gov (accessed on 20 August 2022); study number NCT02688101 and NCT02433626, respectively) [9,10]. Interestingly, in the case of both new TSCs, additional modes of action were suggested, which were associated with the interactions with metal ions other than iron. On the one hand, COTI-2 was suggested to have zinc metallochaperone properties, which results in the restoration of the tumor suppressor activity of certain mutants of the p53 protein, e.g., p53^G245^ [11,12,13]. On the other hand, both DpC and COTI-2 are characterized by the formation of prominently high stability complexes with copper(II), which seem to have additional modes of action, such as the induction of paraptotic cell death [14,15]. This finding not only offers the opportunity to treat diverse tumor entities (by overcoming apoptosis resistance), but also has implications for the resistance profile of these TSCs. In more detail, we demonstrated that acquired resistance to COTI-2 (in contrast to Triapine and the model compound COTI-NH_2_) is based on the formation of a stable copper(II)-TSC-glutathione (GSH) ternary complex, which is recognized and effluxed by the ATP-binding cassette (ABC) transporter ABCC1 [16].

In the study presented here, we tested the impact of two types of terminal NH_2_-disubstitutions on chemical properties and anticancer activity compared to COTI-2 and its terminally non-substituted model compound COTI-NH_2_ (Scheme 1). Namely, an *N*-dimethylated derivative (COTI-NMe_2_, Scheme 1) and a compound with cyclohexyl and methyl substituents (COTI-NMeCy, Scheme 1) were developed. These two structural modifications were selected in analogy to the terminal substitution pattern of DpC and its progenitor Dp44mT. Notably, although COTI-2 is already being used in clinical studies and TSCs are well-known metal chelators, the coordination chemistry of COTI-2 and its derivatives has not been investigated so far. Therefore, we studied the physico-chemical properties of COTI-2 and these new compounds and their complex formation with biologically relevant metal ions in detail. Furthermore, their activity against drug-resistant cancer cells was investigated in comparison with COTI-2 and Triapine. Following this approach, we were able to identify a new derivative that exhibits not only higher anticancer activity, but is also able to overcome acquired resistance to COTI-2 as well as Triapine.

## 2. Materials and Methods

### 2.1. Materials and Methods for Synthesis

COTI-2 and COTI-NH_2_ were prepared as described previously [16]. All solvents and reagents were obtained from commercial suppliers and used without further purification. A Perkin Elmer 2400 CHN Elemental Analyzer at the Microanalytical Laboratory of the University of Vienna was used for elemental analyses. All values were within ±0.4%, confirming >95% purity. Electrospray ionization (ESI) mass spectra were obtained using a Bruker amaZon SL ion trap mass spectrometer in the positive mode by direct infusion, while high-resolution mass spectra were obtained on a Bruker maXis UHR ESI time-of-flight mass spectrometer. One-dimensional ^1^H-NMR spectra of the precursors were recorded on a Bruker Avance III 500 MHz spectrometer at 298 K. One- and two-dimensional ^1^H-NMR and ^13^C-NMR spectra of the final products were recorded on a Bruker Avance III 600 MHz spectrometer at 298 K. For ^1^H-NMR spectra the solvent residual peak was taken as yjr internal reference (s = singlet; d = doublet; t = triplet; quint = quintet; dd = doublet of doublets; ddd = doublet of doublet of doublets; br = broad signal; m = multiplet; py = pyridine). For the NMR numbering of the compounds, see Scheme S1 and Scheme S2.

#### 2.1.1. Synthesis of 2-(6,7-Dihydroquinolin-8(5H)-ylidene)-*N*^4^,*N*^4^-dimethylhydrazine-1-carbothioamide (COTI-NMe_2_)

5,6,7,8-Tetrahydroquinolin-8-one (0.37 g, 2.5 mmol, 1 equiv.) was dissolved in isopropanol (10 mL), followed by the addition of one drop of concentrated HCl. Subsequently, 4,4-dimethyl-3-thiosemicarbazide (0.30 g, 2.5 mmol, 1 equiv.) was added and the mixture was stirred under reflux for 5 h. This led to the formation of a yellow solid, which was filtered off, washed with cold isopropanol as well as diethyl ether, and dried under a vacuum. Yield: 0.45 g (71%). Elemental analysis: Calcd. for C_12_H_16_N_4_S·0.25H_2_O (%): C, 57.00; H, 6.58; N, 22.16; and S, 12.68. Found (%): C, 57.25; H, 6.42; N, 25.53; and S, 12.44. ESI-MS in acetonitrile (can)/methanol (MeOH) + 1% H_2_O (positive): *m*/*z* 271.20, [HL + Na]^+^. Major isomer (67%) ^1^H-NMR (600 MHz, DMSO-*d*_6_; for the numbering see Scheme S1): δ = 1.93 (quint, *^3^J* = 6.3 Hz, 2H, CH_2_, H6), 2.72 (t, *^3^J* = 5.9 Hz, 2H, CH_2_, H7), 2.91 (t, *^3^J* = 5.9 Hz, 2H, CH_2_, H5), 3.27 (s, 6H, N(CH_3_)_2_, H11,12), 7.47 (dd, *^3^J* = 4.8 Hz, *^3^J* = 7.8 Hz, 1H, CH_py_, H2), 7.88 (d, *^3^J* = 7.4 Hz, 1H, CH_py_, H3), 8.62 (dd, *^4^J* = 1.2 Hz, *^3^J* = 4.7 Hz, 1H, CH_py_, H1), and 14.48 (s, 1H, NH, N3). ^13^C NMR (DMSO-d_6_): δ = 22.1 (CH_2_, C6), 28.8 (CH_2_, C5), 33.4 (CH_2_, C7), 40.7 (CH_3_, C11,12), 124.1 (C_py_, C2), 136.0 (C_q_, C4), 138.7 (C_py_, C3), 141.1 (C=N, C8), 145.4 (C_py_ C1), 148.4 (C_q_, C9), and 179.9 (C=S, C10). Minor isomer (33%) ^1^H-NMR (600 MHz,DMSO-*d*_6_): δ = 1.96 (quint, *^3^J* = 6.1 Hz, 2H, CH_2_, H6), 2.88 (t, *^3^J*= 6.1 Hz, 2H, CH_2_, H7), 3.05 (t, *^3^J* = 6.4 Hz, 2H, CH_2_, H5), 3.34 (s, 6H, N(CH_3_)_2_, H11,12), 7.42 (dd, *^3^J* = 4.7 Hz, *^3^J* = 7.8 Hz, 1H, CH_py_, H2), 7.76 (dd, *^3^J* = 7.6 Hz, *^3^J* = 4.5 Hz, 1H, CH_py_, H3), 8.57 (dd, *^4^J* = 1.1 Hz, *^3^J* = 4.5 Hz, 1H, CH_py_, H1), and 15.51 (s, 1H, NH, N3). ^13^C NMR (DMSO-*d*_6_): δ = 20.0 (CH_2_, C6), 26.3 (CH_2_, C5), 27.4 (CH_2_, C7), 40.7 (CH_3_, C11,12), 125.1 (C_py_, C2), 137.1 (C_q_, C4), 137.7 (C_py_, C3), 141.6 (C=N, C8), 148.2 (C_py_ C1), 144.1 (C_q_, C9), and 184.4 (C=S, C10). The ^1^H and ^13^C spectra can be found in Figure S1 and Figure S2, respectively.

#### 2.1.2. Synthesis of *N*-Cyclohexyl-2-(6,7-dihydroquinolin-8(5H)-ylidene)-*N*^4^-methylhydrazine-1-carbothioamide (COTI-NMeCy)

The first two steps (Scheme S3) were performed according to a modified procedure from reference [16].

##### Synthesis of 2-((Cyclohexyl(methyl)carbomothioyl)thio)acetic Acid (**1a**)

*N*-Methylcyclohexylamine (1.3 mL, 10.0 mmol, 1 equiv.) was added to 1 M NaOH (12 mL), resulting in a white suspension. This suspension was stirred, while 0.6 mL of carbon disulfide (10.0 mmol, 1 equiv.) was added dropwise. A yellow precipitate formed, and the mixture was stirred for 20 min. Subsequently, a solution containing sodium chloroacetate (1.16 g, 10.0 mmol, 1 equiv. in 8 mL water) was added. The mixture was stirred overnight at room temperature. On the next day, 0.65 mL of concentrated HCl (7.9 mmol) was added to the yellow solution, and the resulting white precipitate (**2a**) was filtered off, washed with water, and dried under a vacuum. Yield: 1.89 g (76%). ^1^H-NMR (500.10 MHz, DMSO-*d*_6_): δ = 1.16–1.83 (m, 10H, CH_2 cyclohexyl_), 3.20 (s, 3H, NCH_3_), 4.09 (s, 2H, CH_2_), 4.34–5.27 (m, 1H, CH_cyclohexyl_), and 12.78 (br, 1H, OH).

##### Synthesis of *N*-Cyclohexyl-*N*-methylhydrazinecarbothioamide (**1b**)

Precipitate **1a** (1.04 g, 4.23 mmol) was dissolved in hydrazine hydrate (5 mL, 100 mmol) followed by the addition of 2 mL of water. The mixture was stirred at 50–60 °C for 2 h. A white precipitate (**1b**) was formed, which was filtered off, washed with water, and dried under vacuum. Yield: 1.05 g (75%). ^1^H-NMR (500.10 MHz, DMSO-*d*_6_): δ = 1.04–1.76 (m, 10H, CH_2 cyclohexyl_), 2.85 (s, 3H, NCH_3_), 4.70 (s, 2H, NH_2_), 4.85 (bs, 1H, CH_cyclohexyl_), and 8.70 (s, 1H, NH).

##### Synthesis of *N*-Cyclohexyl-2-(6,7-dihydroquinolin-8(5H)-ylidene)-*N*^4^-methylhydrazine-1-carbothioamide (**COTI-NMeCy**)

Precipitate **1b** (0.58 g, 3.12 mmol, 1 equiv.) was suspended in 8 mL of isopropanol at 80 °C, followed by the addition of 5,6,7,8-tetrahydroquinolin-8-one (0.46 g, 3.12 mmol, 1 equiv.). Then, the mixture was stirred under reflux for 3 h. A yellow solid was formed, which was filtered off, washed with cold isopropanol, and dried under vacuum. Yield: 0.59 g (60%). Elemental analysis: Calcd. for C_17_H_24_N_4_S (%): C, 64.52; H, 7.64; N, 17.70; and S, 10.13. Found (%): C, 64.24; H, 7.64; N, 17.65; S, and 9.94. ESI-MS in ACN/MeOH + 1% H_2_O (positive): *m*/*z* 339.1607, [HL + Na]^+^. ^1^H-NMR (600 MHz, DMSO-*d*_6_; for the numbering scheme see Scheme S2): Major isomer (97%) δ = 1.12 (m, 1H, CHchexyl), 1.32 (m, 2H, CHchexyl), 1.50 (m, 2H, CHchexyl), 1.64 (m, 3H, CHchexyl), 1.78 (d, 2H, CHchexyl), 1.96 (quint, *^3^J* = 6.2 Hz, 2H, CH_2_, H6), 2.88 (t, *^3^J* = 6.1 Hz, 2H, CH_2_, H7), 3.05 (m, 5H, CH_2_, H5 + NCH_3_, H17), 4.79–5.09 (bs, 1H, CH, H11), 7.41 (dd, *^3^J* = 4.6 Hz, *^3^J* = 7.8 Hz, 1H, CH_py_, H2), 7.76 (d, *^3^J* = 7.7 Hz, 1H, CH_py_, H3), 8.58 (d, *^3^J* = 4.0 Hz, 1H, CH_py_, H1), and 14.65 (s, 1H, NH, N3). ^13^C NMR (DMSO-*d*_6_): δ = 20.0 (CH_2_, C6), 25.1 (CH_2_, C13,15), 25.4 (CH_2_, C14), 26.2 (CH_2_, C5), 27.2 (CH_2_, C7), 29.5 (CH_2_, C12,16), 30.1 (CH_3_, C17), 58.4 (CH, C11), 125.0 (C_py_, C2), 135.9 (C_q_, C4), 137.7 (C_py_, C3), 144.2 (C_q_, C9), 148.2 (C_py_ C1), 148.4 (C=N, C8), and 183.8 (C=S, C10). ^1^H-NMR (600 MHz, DMSO-*d*_6_): Minor isomer (3%) δ = 4.80 (s, 1H, CH, H11′), 7.48 (dd, 1H, CH_py_, H2′), 7.88 (dd, 1H, CH_py_, H3′), and 15.38 (s, 1H, NH, N3′). ^13^C NMR (DMSO-*d*_6_): δ = 22.1 (CH_2_, C6′), 24.9 (CH_2_, C13,15′), 25.5 (CH_2_, C14′), 28.8 (CH_2_, C7′), 33.4 (CH_2_, C12,16′), 124.1 (C_py_, C2′), 137.1 (C_q_, C4′), 138.7 (C_py_, C3′), and 145.4 (C_py_ C1′). Due to the small amount of the second isomer, not all proton and carbon atoms could be assigned and no coupling constants were calculated. The ^1^H and ^13^C spectra can be found in Figure S3 and Figure S4, respectively.

#### 2.1.3. Synthesis of the Cu(II) Complex of COTI-NMe_2_ (Cu-COTI-NMe_2_)

Cu(II) chloride dihydrate (43 mg, 0.25 mmol) was dissolved in 2.5 mL methanol at 40 °C, followed by the addition of 34 µL of concentrated HCl. Then, a suspension of COTI-NMe_2_ (50 mg, 0.2 mmol) in methanol (1.75 mL) was added dropwise to the copper(II) solution. The mixture was stirred for 2 h. The formed dark green solid was filtered off, washed with cold methanol as well as diethyl ether, and dried under a vacuum. Yield: 39 mg (54%). Elemental analysis: Calcd. for [Cu(C_12_H_15_N_4_S)Cl]·0.25H_2_O (%): C, 41.08; H, 4.45; N, 15.97; and S, 9.14. Found (%): C, 41.12; H, 4.26; N, 15.72; and S, 9.32. ESI-MS in ACN/MeOH + 1% H_2_O (positive): *m*/*z* 310.03, [M-Cl]^+^; 657.03, [2M-Cl]^+^.

#### 2.1.4. Synthesis of the Cu(II) Complex of COTI-NMeCy (Cu-COTI-NMeCy)

Cu(II) chloride dihydrate (34 mg, 0.20 mmol, 1.25 equiv.) was dissolved in methanol (2.5 mL) at 40 °C, followed by the addition of 34 µL of concentrated HCl. Next, a suspension of COTI-NMeCy (50 mg, 0.16 mmol, 1 equiv.) in methanol (1.75 mL) was added dropwise to the Cu(II) solution. The mixture was then stirred for 2 h. Overnight, dark green crystals formed in the solution, which were filtered off, washed with cold methanol as well as diethyl ether, and dried under a vacuum. Yield: 30 mg (45%). Elemental analysis: Calcd. for [Cu(C_17_H_23_N_4_S)Cl]·0.25 H_2_O (%): C, 48.74; H, 5.65; N, 13.37; and S, 7.65. Found (%): C, 48.36; H, 5.42; N, 13.74; and S, 7.61. ESI-MS in ACN/MeOH + 1% H_2_O (positive): *m*/*z* 378.13, [M-Cl]^+^, and 793.21, [2M-Cl]^+^.

### 2.2. Materials and Methods for Spectrophotometric Investigations

4-(2-Hydroxyethyl)-1-piperazineethanesulfonic acid (HEPES), 2-(*N*-morpholino)ethanesulfonic acid (MES), ascorbic acid (AA), GSH, and tetrabutylammonium nitrate (TBAN) were purchased from Sigma-Aldrich and used without further purification. KOH, KCl, HCl, ethylenediaminetetraacetic acid (EDTA), and KH-phthalate were obtained from Reanal (Hungary) in puriss quality. Cu(II), Zn(II), and Fe(III) stock solutions were prepared by dissolving CuCl_2_, ZnCl_2_, and FeCl_3_ in water (or in a known amount of HCl solution for Zn(II) and Fe(III)) and complexometry with EDTA was used to determine the exact concentrations. To prepare the Fe(II) stock solution, iron powder was dissolved in an HCl solution under an oxygen-free argon atmosphere. After the dissolution of iron, the solution was filtered, stored, and used under anaerobic conditions. The exact concentration of the Fe(II) stock solution was determined by permanganometric titrations. Milli-Q water was used for sample preparation. For EDTA competition measurements, pH 6 was adjusted with 50 mM MES buffer; for the redox reactions with GSH and AA, the pH of the solutions was set to 7.4 with 50 mM of HEPES. All stock solutions of the reducing agents were prepared freshly before each series of measurements.

### 2.3. UV-Visible Spectrophotometric Titrations

The UV-vis spectra were recorded using an Agilent Cary 8454 diode array spectrophotometer in the 200 to 950 nm window, and the path length was varied between 1 and 5 cm, depending on the solubility (thus the applicable concentration). For the spectrophotometric titrations, the same instruments (Metrohm 665 Dosimat burette, Orion 710A pH-meter, Metrohm combined pH glass electrode) and experimental setup (ionic strength: 0.10 M (KCl); titrant: 0.10 M carbonate-free KOH solution in 30% (*v/v*) DMSO/H_2_O; samples: prepared in 30% (*v/v*) DMSO/H_2_O; calibration: method of Irving et al. [17]; pH range: 1.0 to 13.5; and sample volume: 10 cm^3^) were used as in our previous work [15]. Argon overpressure was used for the Fe(II)-containing samples when the Fe(II) stock solution was added to the samples in tightly closed titration vessels, which were previously completely deoxygenated (for 20 min). Measurements were taken with ligands (10–80 μM, depending on solubility) in the presence of 1, 0.5, and 0.3 equiv. of Cu(II), Fe(II), and Fe(III) ions. Equilibrium constants, such as the proton dissociation constants (p*K*_a_) of the ligands, the overall stability constants (log*β*) of the metal complexes, and the UV-vis spectra of the individual ligand or complex species were computed using the program PSEQUAD [18], as was the case in our previous works [19,20,21].

The conditional stability constants (*β*’) of the Cu(II) complexes were calculated from competition reactions with EDTA at pH = 5.9 using MES (50 mM) when the metal:ligand ratio was 1:1 (*c*_lig_ = *c*_Cu_ between 10 and 80 μM). Individual samples were prepared in which the ligand:EDTA ratio was variable between 1:0 and 1:50. After a 3 h incubation period, UV-vis spectra were collected. The calculation of the *β*’ (CuL) values was performed following the same method as that in our previous work [15] using the log*β*’_5_._90_ = 13.89 of complex [Cu(EDTA)]^2−^ obtained from the previously reported equilibrium constants of EDTA and its Cu(II) complex [22].

### 2.4. Electron Paramagnetic Resonance Measurements and Evaluation of the Spectra

The anisotropic electron paramagnetic resonance (EPR) spectra were recorded using a BRUKER EleXsys E500 spectrometer (microwave power 13 mW, microwave frequency 9.43 GHz, modulation amplitude 5 G, and modulation frequency 100 kHz, at 77 K). The spectra were evaluated individually with the “EPR” program [23] following the same approach as that in our previous works [19,20]. More details regarding the sample compositions and the calculations can be found in the Supplementary Materials.

### 2.5. Spectrophotometric Kinetic Measurements

A Hewlett Packard 8452A diode array spectrophotometer and a special, tightly closed tandem cuvette (Hellma Tandem Cell, 238-QS) were used to monitor the redox reactions of the Cu(II) and Fe(III) complexes with GSH and AA. The spectra were recorded at a physiological pH (pH = 7.4, 50 mM HEPES, *I* = 0.1 M KCl) when the metal complexes:GSH or AA ratio was 1:300 (*c*_complex_ = 30 μM, *c*_GSH or AA_ = 9 mM). The stock solutions of the complexes and the GSH and AA solutions were freshly prepared before every measurement. In the first half of the cuvette, the ligand was mixed with the complex, and the other contained the reducing agent. Both isolated parts of the cuvette were deoxygenated with argon for 10 min. The first spectrum was recorded before mixing, and then after mixing, the reduction was usually followed until no further absorbance change was observed.

### 2.6. Lipophilicity, Solubility, and Artificial Membrane Permeability Assays

Distribution coefficient (*D*_7_._4_) values of COTI-NH_2_ were determined by the shake-flask method in *n*-octanol/buffered aqueous solution at pH 7.4 in 20 mM phosphate buffer (0.10 M KCl) at 25.0 ± 0.2 °C following a similar approach to that reported in our previous works [8,20]. The other compounds and all of their Cu(II) complexes were too lipophilic to obtain experimental data. Thus, the log*P* values were calculated for COTI-NMeCy, COTI-Nme_2_, and COTI-2 (MarvinSketch,version 16.12.12.0, calculation module developed by ChemAxon (Budapest, Hungary), http://www.chemaxon.com/products/marvin/marvinsketch/ (accessed on 20 August 2022)).

The thermodynamic solubility of COTI-NMeCy, COTI-Nme_2_, COTI-NH_2_, and COTI-2 was measured for the saturated solutions in water at pH 7.4 (20 mM HEPES buffer) at 25.0 ± 0.1 °C. The concentrations of the compounds were determined by UV-vis spectrophotometry using stock solutions of the compounds with known concentrations dissolved in pure DMSO, and 50% and 1% (*v/v*) DMSO/buffered aqueous solution for the calibration. The parallel artificial membrane permeability assay (PAMPA) was applied for the ligands with a Corning Gentest pre-coated PAMPA Plate System [24]. *P*_eff_ values were calculated according to the equation reported by Yu et al. [25].

### 2.7. Cyclic Voltammetry

Cyclic voltammograms for the Fe(III) and Cu(II) complexes were recorded in 90% (*v/v*) DMSO/water solution containing 1 mM ligand and 0.5 mM FeCl_3_ or 1 mM CuCl_2_ using a conventional three-electrode system under nitrogen atmosphere and a PC-Controlled Electrochemical Measurement System (EF 451). The ionic strength was 0.10 M, adjusted by TBAN. Samples were purged for 15 min with Ar before recording the cyclic voltammograms. Platinum electrodes were used as the working and auxiliary electrodes, and Ag/AgCl (3 M KCl) was applied as the reference electrode. The electrochemical potentials were converted into the normal hydrogen electrode (NHE) scale by adding 0.222 V. The instrument was calibrated with an aqueous solution of K_3_[Fe(CN)_6_] (E_1/2_ = +0.386 V vs. NHE). Redox potentials were obtained at different scan rates (5–25 mV/s) in the range of −0.8 to +0.1 V. The dependence of the peak current values of the square root of the scan rates is shown in Figure S5 for the iron and copper complexes of COTI-NMeCy.

### 2.8. Cell Culture Studies

In vitro tests were performed with human colorectal cancer SW480 cells (purchased from the American Type Culture Collection, Manassas, VA, USA), SW480/Tria, and the SW480/Coti cells, which are previously established sublines that are resistant to Triapine [26] and COTI-2 [16], respectively. Additionally, small-cell lung carcinoma GLC-4 and its subline GLC-4/adr, which is a doxorubicin-resistant subline overexpressing ABCC1 (purchased from Dr. deVries, Groningen, The Netherlands), were used. All cells were maintained in a humidified 5% CO_2_ incubator at 37  °C and grown in minimal essential medium or RPMI-1640, respectively, supplemented with 10% fetal bovine serum (FBS). In order to maintain the resistant phenotype of GLC-4/adr cells, they were selected with doxorubicin (1152 nM) once a month. All cell lines were tested for *mycoplasma* contamination.

### 2.9. Viability Assays

For the cytotoxicity, cell viability, and proliferation studies, the 3-(4,5-dimethylthiazol-2-yl)-2,5-diphenyltetrazolium bromide (MTT) assay was utilized. Cells were seeded in 96-well plates at a density of 2 × 10^4^ cells/well, with the exception of SW480/Tria, which was used at a density of 3 × 10^4^ cells/well. After 24 h of recovery, cells were treated with the indicated concentration of drugs. For the drug combination experiments, verapamil and cyclosporine A (CSA) were used as ABCC1 modulators, and tetrathiomolybdate (TTM) was used as a copper chelator. The TSCs were dissolved in DMSO followed by subsequent dilutions in the medium in order to maintain a final DMSO concentration of <1%. After 72 h, the treatment solution was removed and replaced with 100 µL of MTT solution (EZ4U, Biomedica, Vienna, Austria), which was diluted 1:10 in the growth medium. A TECAN Infinite 200 Pro plate reader was used to measure the absorbance of the solution after 2–3 h incubation at 5% CO_2_ in an incubator at 37  °C. The anticancer activity was determined with GraphPad Prism software and indicated as IC_50_ values (half maximum inhibitory concentration).

### 2.10. Cell Death Analysis by Flow Cytometry

In the following staining protocol, annexin V (AV) was used to label exposed phosphatidylserine, an early cell death marker, and propidium iodide (PI) to stain dead cells. SW480 or SW480/Coti cells were seeded at a density of 2 × 10^5^ per well in 6-well plates and treated with the indicated concentrations of COTI-2 and COTI-NMe_2_ for 16, 24, or 48 h. Subsequently, the medium was collected and adherent cells were collected by trypsinization. Then, the cells were stained with allophycocyanine-labeled AV (BD Biosciences, San Jose, CA, USA) and PI (0.01 mg/mL, Sigma, San Jose, CA, USA) as previously described [27]. Stained cells were subjected to flow cytometry (BD LSRFortessa, BD Austria GmbH, Vienna, Austria) and analyzed using FlowJo V10 software (BD Life Sciences, Ashland, CA, USA).

### 2.11. Cell Death Analysis by DAPI Staining

In order to detect mitotic nuclei, fixed cells were stained with DAPI. SW480 and SW480/Coti cells were seeded at a density of 2 × 10^5^ cells per well in 6-well plates and left to recover for 24 h. This was followed by drug treatment at the indicated concentrations for 24 h. Then, cells were collected and spun on slides by centrifugation with a Thermo Scientific Cytospin 4 (400 rpm, 5 min at 4 °C). The slides were dried, and the cells were fixed with a methanol/acetone (1:1) mixture at −20 °C and stained with a solution containing DAPI (1 µg/mL). A final washing step with phosphate-buffered saline (PBS) was performed, followed by covering the slides with vectashield (H-100, Vector Laboratories, Inc., Newark, CA, USA) and coverslips. Mitotic nuclei were counted with Image J in three different areas per slide from three independent experiments.

### 2.12. Cell Cycle Analysis by Flow Cytometry

The cell cycle phases were analyzed by staining the DNA of ethanol-fixed cells with PI, as previously described [27]. SW480 and SW480/Coti cells were seeded at a density of, 2 × 10^5^ cells/well in 6-well plates and treated with the indicated concentrations of COTI-2 and COTI-NMe_2_ for 24 h. Then, cells were collected by trypsinization and fixed in cold 70% ethanol. In order to digest RNA, the cells were treated with RNase (0.2 mg/mL, Sigma) and, subsequently, DNA was stained with PI (1 µg/mL). Fluorescence was measured by flow cytometry (FACS Calibur, Becton Dickinson, Ashland, CA, USA) and cell cycle phases were analyzed using Cell Quest Pro software.

### 2.13. Detection of Paraptotic Vesicles

SW480 and SW480/Coti cells were seeded at a density of 2 × 10^4^ per well in 24-well plates and left to recover for 24 h. This was followed by drug treatment at the indicated concentration for 24 h. Images were captured with phase contrast using a Nikon eclipse Ti-e fluorescence microscope and a DS Fi1c camera. Cells containing vesicles were counted in ImageJ and plotted in GraphPad Prism as the percentage of all cells.

### 2.14. HPLC-MS Cell Uptake Measurements

Cells (1 × 10^6^) were seeded in 25 cm^2^ culture flasks and left to recover for 24 h at 37 °C and 5% CO_2_. The compounds were dissolved in DMSO (10 mM) and further diluted in the growth medium. The cells were treated in triplicate by replacing the growth medium with 5 mL of drug-containing medium (10 µM). After 3 h of incubation the cells were washed once with ice-cold PBS, and then collected by scraping into ice-cold PBS. The cell suspension was centrifuged for 5 min at 300× *g* and the supernatant was removed. Then, the cell pellets were lysed with three freeze/thaw cycles in liquid nitrogen/37 °C waterbath and re-suspended in 100 µL of water and 300 µL of cold acetonitrile (1:3 mixture). After 10 min of incubation on ice, the samples were centrifuged (15,080× *g*, 4 °C, and 10 min) and the supernatants were stored at −80 °C for HPLC-MS analysis. For the calibration curve, known concentrations of drugs were spiked into untreated cell extracts before adding acetonitrile. To normalize to the cell number, additional flasks were prepared (treated and untreated) and cells were collected by trypsinization and counted with counting chambers.

LC-MS analyses of the cell and media extracts were performed on a Vanquish Horizon UHPLC system (Thermo Fisher Scientific, Waltham, MA, USA) coupled to the ESI source of a timsTOF fleX mass spectrometer (Bruker Daltonics, Billerica, MA, USA). Separation was carried out on an Acquity UPLC HSS T3, 2.1 × 50 mm, 1.8 µm HPLC column (Waters) using water (mobile phase A) and methanol (mobile phase B). Both mobile phases were modified with 0.1% formic acid. High-resolution ESI-MS spectra were recorded using the positive ion mode in the range of *m*/*z* 150–510 at a spectra rate of 2 Hz. The ESI ion source settings were as follows: capillary voltage: 4.5 kV, nebulizer: 2.2 bar (N_2_), dry gas flow: 10.0 L/min (N_2_), and dry temperature: 220 °C. For quantification, extracted ion chromatograms of *m*/*z* 249.1168 ± 0.0020 for COTI-NMe_2_ and of *m*/*z* 367.1699 ± 0.0020 for COTI-2 were calculated and the peak areas were determined using Bruker Compass Data Analysis 5.3.

In general, it must be mentioned that the LC-MS method for quantification of COTI-2 and COTI-NMe_2_ was not validated and that the deviation of the peak areas both within a batch and between batches was higher than expected. Thus, the absolute values for the intracellular drug levels should be considered with care and not be over-interpreted. However, two independent experiments were performed, revealing good accordance, and similar trends were also observed in a third scouting experiment without calibration.

## 3. Results and Discussion

### 3.1. Syntheses and Chemical Characterization

Two novel COTI-2 derivatives were synthesized with modified terminal *N*^4^-substitutions, namely the simple *N*-dimethyl (COTI-NMe_2_) compound and the *N*-methyl-cyclohexyl-substituted derivative (COTI-NMeCy) in analogy to the amino moiety of DpC (Scheme 1). For COTI-NMeCy, the *N*^4^-moiety was formed by the reaction of carbon disulfide with *N*-methyl-cyclohexylamine and sodium chloroacetate (Scheme S3). After the reaction with hydrazine hydrate, the generated thiosemicarbazide was treated with 5,6,7,8-tetrahydroquinoline-8-one to yield COTI-NMeCy. COTI-NMe_2_ was obtained by the Schiff base condensation reaction of the ketone with the appropriate commercially available *N*^4^-Me_2_ thiosemicarbazide.

**Scheme 2:**
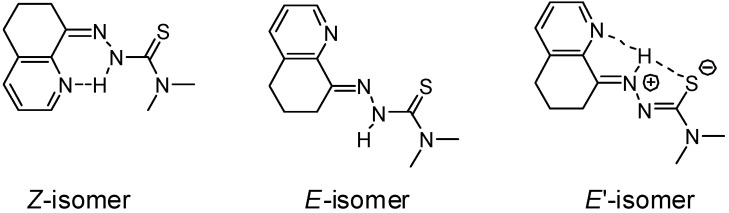
Different possible isomers of COTI-NMe_2_.

The ^1^H NMR spectra of COTI-NMe_2_ and COTI-NMeCy (Figures S1 and S3) indicated the presence of two isomers in the applied solvent (DMSO-*d*_6_), a phenomenon well-known for TSCs [26,27]. Interestingly, for both compounds, a chemical shift of the hydrazonic-NH proton for both isomers was found in the range of 14.3–15.5 ppm. Consequently, we assigned them to the *Z*- and *E*′-isomers out of the three possible structures (see Scheme 2 for COTI-NMe_2_), which both possess an intramolecular hydrogen bond explaining the strong downfield shift. The same pattern was also observed in the NMR spectrum of COTI-2 [16]. In contrast, the *E*-isomer (without a hydrogen bond) usually has an NH signal resonance at around 9–10 ppm [26,27]. Additionally, the X-ray crystal structure of COTI-2 [16] revealed the zwitterionic *E*’-isomer in which the imine N2 was protonated, generating a positive charge, and the C–S single bond forming the negative charge.

### 3.2. Characterization of the Solution Chemical Properties

On the one hand, the solution chemical properties, such as protonation state, solubility, and lipophilicity, can strongly affect the pharmacokinetics of a drug molecule. On the other hand, for the interpretation of biological data, the knowledge of the actual chemical form of a drug molecule in aqueous solution, especially at a physiological pH, is highly important. Therefore, acidity (proton dissociation) constants (p*K*_a_), thermodynamic solubility (*S*), and distribution coefficients (*D*_7.4_) were determined.

**Figure 1:**
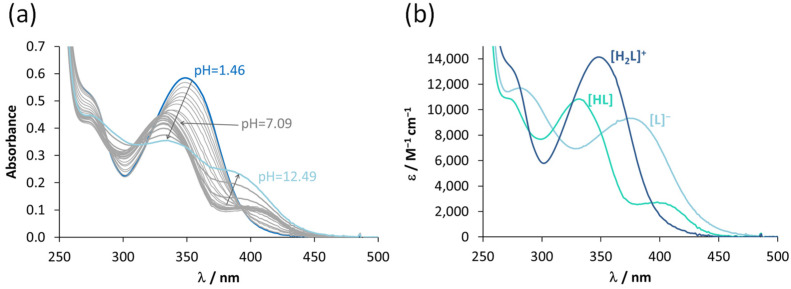
(**a**) UV*-*vis spectra of COTI-NMeCy recorded at various pH values, and (**b**) calculated individual molar absorption spectra for the species in the different protonation states. *c_compound_* = 20 µM; pH = 1.5–12.5; *T* = 25 °C; *I* = 0.10 M (KCl); *ℓ* = 1.0 cm; and 30% (*v*/*v*) DMSO/H_2_O.

The proton dissociation processes of COTI-2, COTI-NH_2_, COTI-NMe_2_, and COTI-NMeCy were monitored by UV-visible (UV-vis) spectrophotometric titrations. As the compounds had limited water solubility, the titrations were performed at low concentrations (10–80 μM) in a 30% (*v/v*) DMSO/H_2_O solvent mixture in the pH range between 1.0 and 13.5, similar to those reported for related TSCs in our previous works [19,28]. COTI-NH_2_, COTI-NMe_2_, and COTI-NMeCy are considered as diprotic compounds (H_2_L^+^) with the deprotonating moieties dihydroquinolinium-NH^+^ and hydrazone-NH. In contrast, COTI-2 contains an additional dissociable proton, namely pyridinium-NH^+^. Representative spectra for COTI-NMeCy are shown in Figure 1, and those for the other compounds are shown in Figure S6. UV-vis spectrophotometric titrations revealed characteristic spectral changes in the 250–450 nm wavelength range for all compounds upon changing the pH.

Based on the UV-vis spectra, p*K_a_* values (Table 1) and individual molar absorption spectra were calculated for the species in differently protonated forms (Figure 1b and Figure S6). Due to the fairly similar structures and the observed spectral changes in the diprotic COTI-NMe_2_, COTI-NMeCy, and COTI-NH_2_ compared with those of Triapine [16], the p*K*_a_ (H_2_L) values can be attributed to the proton dissociation of the dihydroquinoline unit, and p*K_a_* (HL) to the hydrazone NH group of the thiosemicarbazide moiety. In the case of COTI-2, p*K*_a_ (H_3_L), p*K*_a_ (H_2_L), and p*K*_a_ (HL) were assigned to the deprotonation of dihydroquinolinium–NH^+^, pyridinium–NH^+^, and hydrazonic–NH moieties, respectively. Based on the determined p*K_a_* values, concentration distribution curves were calculated (Figure S7) to represent the pH range for the formation of the species in the different protonation states. We can conclude that, in solution, all the studied compounds were present in their neutral HL form in the physiological pH range, as depicted in Scheme 1.

As a next step, the thermodynamic solubility of the TSCs was characterized in water at pH 7.4 using UV-vis spectrophotometry for the analysis. The determined solubility (*S*) values are collected in Table 1 and compared with predicted data obtained by a SwisssADME tool (http://www.swissadme.ch; accessed on 20 August 2022). Both approaches showed the same trend in solubility: COTI-2 < COTI-NMeCy << COTI-NMe_2_ < COTI-NH_2_. To characterize the lipophilicity of the compounds, distribution coefficients (*D*_7.4_) were determined using *n*-octanol/water partitioning at pH 7.4. However, experimental data could only be obtained for COTI-NH_2_, characterized by the highest solubility and lowest lipophilicity. For the other compounds, only the constants were calculated (Table 1). The lipophilicity trend was approximately consistent with the solubility of the compounds (Table 1). All COTI-2 derivatives were more lipophilic than Triapine (log*D*_7.4_ = +0.85) [29]. In addition, PAMPA was performed to monitor the ability of the compounds to penetrate cell membranes by passive diffusion (the proposed mode of cell entry). UV-vis spectra recorded for the donor and acceptor phases allowed the calculation of the effective passive permeability coefficients (*P*_eff_, Table 1) for COTI-NH_2_ and COTI-NMe_2_ at pH 7.4 (Figure S8), while precipitation in the case of COTI-2 and COTI-NMeCy hindered their measurement. The *P*_eff_ values of COTI-NH_2_ and COTI-NMe_2_ indicate their high membrane permeability (*P*_eff_ ≥ 1.5 × 10^−6^ cm/s); thus, the more lipophilic COTI-2 and COTI-NMeCy are assumed to possess even higher *P*_eff_ values.

### 3.3. Interaction of the COTI-2 Derivatives with Iron(II) and Iron(III) Ions

As already mentioned in the introduction section, interaction with biologically relevant metal ions is crucial for the anticancer activity of α-*N*-heterocyclic TSCs. However, depending on their exact chemical structure, either iron or copper seems to be the major player [4]. Furthermore, for COTI-2, zinc was suggested as an intracellular target, as it is essential for the proper folding of the zinc-binding protein and transcription factor p53 [11,12]. Therefore, the pH-dependent stoichiometry and the overall stability constants were determined for the iron(III), iron(II), copper(II), and zinc(II) complexes of COTI-2, COTI-NH_2_, COTI-NMe_2_, and COTI-NMeCy.

**Figure 2:**
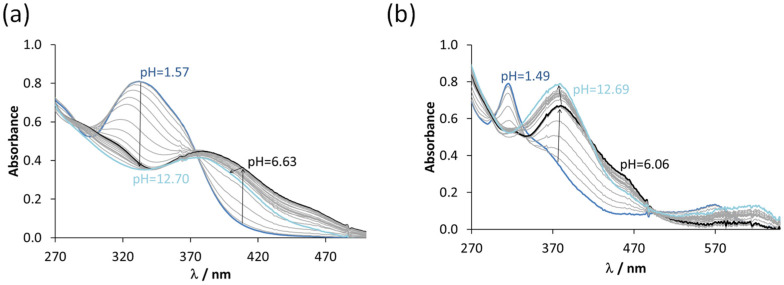
UV*-*vis spectra of the (**a**) iron(III)–COTI-2 (1:2) and (**b**) iron(II)–COTI-NMe_2_ (1:2) systems recorded at various pH values; (**a**): *c*_L_ = 20 µM and *c*_Fe(III)_ = 10 µM; (**b**): *c*_L_ = 30 µM, *c*_Fe(II)_ = 15 µM, *T* = 25 °C, *I* = 0.10 M (KCl), *ℓ* = 2.0 cm, and 30% (*v/v*) DMSO/H_2_O.

For the iron(II/III) complexes, UV-vis spectrophotometric titrations in 30% (*v/v*) DMSO/water were performed (strictly anaerobic conditions were used for iron(II)). Representative UV-vis spectra of the iron(III)–COTI-2 and iron(II)–COTI-NMe_2_ systems are shown in Figure 2. The determined stability constants obtained by the deconvolutions of the spectra are presented in Table 2. The studied TSCs formed mono- and bis-ligand complexes with both iron ions similarly to other related tridentate α-*N*-pyridyl-TSCs, such as Triapine and its derivatives [20]. In these complexes, the coordination of the deprotonated ligands via the {N,N,S^−^} donor set was assumed. This binding mode reported for the iron(II) complexes of α-*N*-pyridyl-TSCs was accompanied by the development of an absorption band in the visible wavelength range (~615 nm) [20]. The spectra recorded for the iron(II) complexes of COTI-NH_2_, COTI-NMe_2_ (Figure 2b), COTI-NMeCy, and COTI-2 revealed similar bands. In the case of COTI-2, protonated complexes, such as [Fe(III)LH]^3+^, [Fe(II)LH]^2+^, [Fe(II)L_2_H_2_]^2+^, and [Fe(II)L_2_H]^+^, were also formed, in which the proton belonged to the non-coordinating pyridinium-nitrogen. The representative concentration distribution curves calculated using the overall stability constants (Figure S9) reveal that the mono-ligand and the protonated complexes were formed in the acidic pH range, whereas the bis-ligand [Fe(III)L_2_]^+^ and [Fe(II)L_2_] species dominated at pH > 6 in all the studied systems. In order to compare the iron(II)- and iron(III)-binding abilities of the studied compounds, pFe values were computed at pH 7.4 (Table 2). pFe is the negative decadic logarithm of the equilibrium concentration of the free (unbound) metal ion. Thus, higher values indicate higher solution stability under the given conditions. Among the studied ligands, COTI-2 formed the highest stability complexes with iron(II) (pFe = 19.3), while COTI-NMe_2_ had the highest iron(III)-binding ability (pFe = 19.2; pFe* = 11.1 referring to all of the unbound forms of iron(III), including the hydroxido species), while for iron(II), the pFe was only =17.8, which was the range of those of COTI-NH_2_ and COTI-NMeCy (pFe = 17.4 and 16.9).

**Figure 3:**
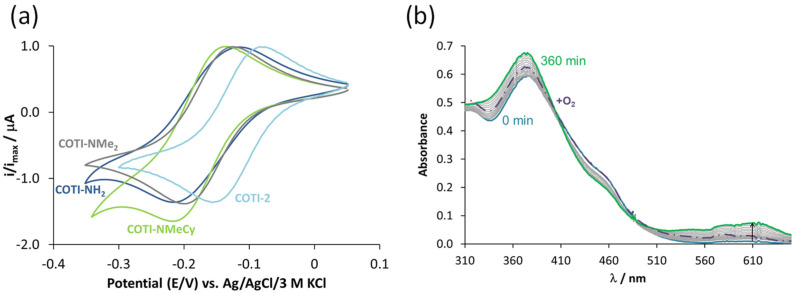
Cyclic voltammograms and interaction with glutathione (GSH) of iron(III)–thiosemicarbazone complexes. (**a**) Normalized cyclic voltammograms of the iron(III)–thiosemicarbazone (1:2) systems at a 0.01 V/s scan rate. *c*_L_ =1 mM, *c*_Fe_ = 0.5 mM, pH = 7.40 (10 mM HEPES), *T* = 25 °C, *I* = 0.1 M (TBAN), and 90% (*v/v*) DMSO/H_2_O. (**b**) Time-dependent changes in the UV-vis absorption spectra of the iron(III)–COTI-NMe_2_ (1:2) system in the presence of 300 equiv. GSH (solid lines) at pH 7.4 in 5% (*v/v*) DMSO/H_2_O under argon and the spectrum of the free ligand (dashed line). *c*_Fe(III)_ = 30 µM, *c*_L_ = 60 µM, *c*_GSH_ = 9 mM, *T* = 25 °C, *I* = 0.1 M (KCl), and *ℓ* = 1.0 cm.

To monitor the redox properties of the iron complexes, cyclic voltammetric studies were performed as a first step. The cyclic voltammograms revealed reversible redox processes (Figure 3a) due to the presence of bis-ligand complexes in both oxidation states; consequently, reduction and re-oxidation did not disturb the coordination sphere. The calculated formal potentials (Table 2 and Table S6) were in the range of those of the iron complexes of other α-*N*-pyridyl-TSCs (e.g., Triapine (*E′* = +0.07 V) and their *N*-terminally dimethylated derivatives (*E′* = +0.05 V) [20]). The formal potentials were similar for the complexes of COTI-NH_2_, COTI-NMe_2_, and COTI-NMeCy (*E′* = +0.05–0.06 V vs. NHE), whereas a somewhat higher value was obtained for COTI-2 (*E′* = +0.11 V vs. NHE). This finding confirms the slightly higher preference of COTI-2 for iron(II) already visible in the pFe values.

Finally, the direct reduction of the iron(III) complexes by GSH and AA was also followed. Upon addition, for both reducing agents, the typical absorption bands of the iron(II) complexes in the 500–650 nm range appeared (see the spectra for the iron(III)–COTI-NMe_2_–GSH system in Figure 3b). Bubbling O_2_ into the samples resulted in a slow decrease in these bands, showing that the iron(II) complexes could be re-oxidized, although not completely. The reaction was relatively slow in all cases, and the behavior of the tested iron(III) complexes was fairly similar. No significant differences were observed in the reaction rates with the given reducing agents. Generally, the reaction with GSH was twice as fast as that with AA.

### 3.4. Interaction of the COTI-2 Derivatives with Copper(II) and Zinc(II)

As we recently discovered that the copper chemistry has a profound impact on the biological behavior of TSCs, including COTI-2 (e.g., drug resistance mechanisms and excretion) [8,15,16], the interaction of COTI-2 and its new derivatives with copper(II) was studied in detail. To this end, complex formation equilibrium processes with copper(II) ions were characterized by UV-vis spectrophotometric titrations in a 30% (*v/v*) DMSO/water mixture. Titrations performed at ligand excess indicated that (in contrast to iron ions) no bis-complex formation took place, and only mono-ligand species were formed. The spectra recorded at pH 1 in an equimolar solution of the ligands and the metal ion already showed significant complex formation, indicating high-stability complexes.

**Figure 4:**
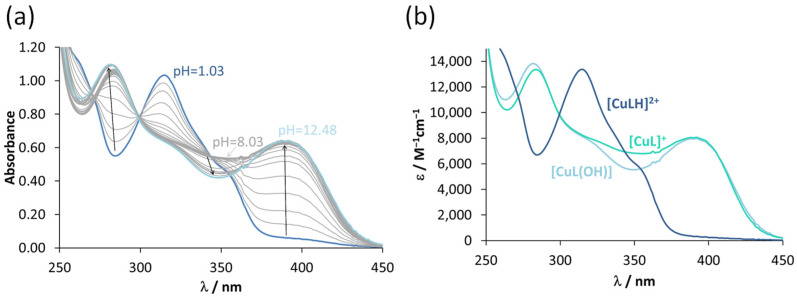
(**a**) UV*-*vis spectra of the copper(II)–COTI-NH_2_ (1:1) system recorded at various pH values and (**b**) calculated individual molar absorption spectra; *c*_Cu(II)_ = *c*_L_ = 80 µM, pH = 1.0–12.5, *T* = 25 °C, *I* = 0.10 M (KCl), *ℓ* = 1.0 cm, and 30% (*v/v*) DMSO/H_2_O.

The spectra for the copper(II)–COTI-NH_2_ (1:1) system revealed two well-separated processes in two pH ranges (ca. 1–3 and 8–10; Figure 4a). Based on these spectral changes, two p*K*_a_ values could be computed (Table 3). The calculated molar absorbance spectra (Figure 4b) and the p*K*_a_ values were very similar to those reported for the copper(II) complexes of Triapine [19], suggesting a similar speciation. Namely, [CuLH]^2+^ was formed in the acidic pH range in which the neutral ligand coordinated. [CuL]^+^ was predominant in a wide pH range (pH~3–8) and the ligand was completely deprotonated due to the dissociation of the hydrazonic NH upon binding. [CuL(OH)], which is a mixed-hydroxido species, was present in the basic pH range. COTI-NMe_2_ and COTI-NMeCy behaved similarly (see p*K*_a_ values in Table 3).

However, the formation of the mixed hydroxido species was accompanied by such minor spectral changes parallel to the appearance of some precipitates that hindered the accurate determination of the p*K*_a_ of [CuL]^+^. For the copper(II) complexes of COTI-2, only one p*K*_a_ (4.66) value could be determined spectrophotometrically in the studied pH range, which was assigned to the deprotonation of the non-coordinating pyridinium-NH^+^ group. In all cases, [CuL]^+^ was the predominant species at a physiological pH.

The high stability of these complexes did not allow us to calculate the overall stability constants (log*β*) directly from the spectra recorded at the different pH values. Thus, EDTA displacement experiments were carried out at pH 5.9, similar to our previous work [15]. A significant decrease in the absorbance was observed in the spectra, where the transition belonged to the characteristic S → Cu^2+^ charge transfer band (λ > 350 nm) upon the addition of the metal chelator EDTA (see Figure S10 for the copper(II)–COTI-NH_2_–EDTA system), indicating a ligand exchange. Using the conditional stability constants (log*β_5.9′_*) for the [CuL]^+^ complexes and the different p*K*_a_ values, the overall stability constants (log*β*) for the various copper(II) complexes were calculated (Table 3). These data revealed that the complexes of all four COTI-2 derivatives were much more stable (log*β* [CuL]^+^ at 16.9–19.1) than Triapine (log*β* [CuL]^+^ at 13.9 [19]), and the following stability trend was obtained: Triapine << COTI-NH_2_ < COTI-NMe_2_ < COTI-NMeCy < COTI-2. Thus, also within the COTI-series, the *N^4^*-terminal di-substitution significantly increased the stability.

In order to obtain information about the coordination modes in these complexes, EPR spectroscopic titrations were performed for the complexes of COTI-NH_2_ and COTI-2. The pH-dependent spectra recorded at 77 K for the copper(II)–COTI-NH_2_/COTI-2 equilibrium systems could be described with three component spectra that could be assigned to [CuLH]^2+^, [CuL]^+^, and [CuL(OH)] (Figure S11). The determined anisotropic EPR parameters are shown in Table S2 and the calculated isotropic *g*_o_, *A*_o_ values are shown in Table S3, and were compared with those of the Triapine complexes [19]. Based on the similarities, the coordination modes {N,N,S}{H_2_O}, {N,N,S^−^}{H_2_O} and {N,N,S^−^}{OH^−^} were suggested in the [CuLH]^2+^, [CuL]^+^ and [CuL(OH)] complexes of COTI-NH_2_, respectively, in solution (where the coordinated H_2_O can be partly replaced by the solvent DMSO). The EPR parameters obtained for the [CuL]^+^ and [CuL(OH)] complexes of COTI-2 were very similar to those of the COTI-NH_2_ complex; only [CuLH]^2+^ slightly differed (for more details, see the additional text in the Supporting Information). The same binding patterns were also assumed for the complexes of COTI-NMeCy and COTI-NMe_2_. Additionally, the coordination of the neutral ligand in the COTI-NH_2_ complex [CuLH]^2+^ could be confirmed by X-ray crystallography (Figure S12). All details can be found in the Supporting Information, including the crystal packing, selected bond lengths, angles, and crystal data (Figures S13–S15, Tables S4–S6).

**Figure 5:**
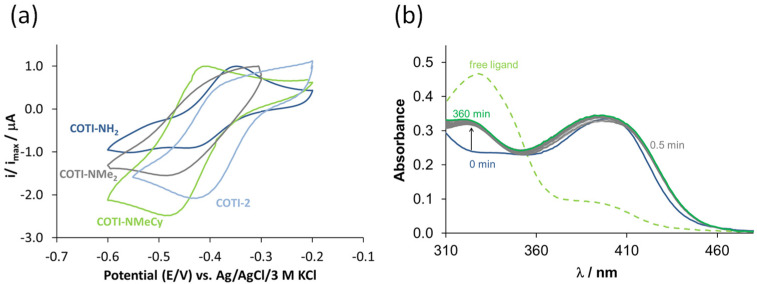
Cyclic voltammetry and interaction with GSH for copper(II)–thiosemicarbazone complexes. (**a**) Normalized cyclic voltammograms of the copper(II)–thiosemicarbazone (1:1) systems at a 0.01 V/s scan rate. *c*_L_ =1 mM, *c*_Cu_ = 1 mM, pH = 7.40 (10 mM HEPES), *T* = 25 °C, *I* = 0.1 M tetrabutylammonium nitrate, and 90% (*v/v*) DMSO/H_2_O. (**b**) Time-dependent changes in the UV-vis absorption spectra of the copper(II)–COTI-NMe_2_ (1:1) system in the presence of 300 equiv. GSH (solid lines) at pH 7.4 in 5% (*v/v*) DMSO/H_2_O under argon and the spectrum of the free ligand (dashed line). *c*_L_ = *c*_Cu(II)_ = 30 µM, *c*_GSH_ = 9 mM, *T* = 25 °C, *I* = 0.1 M (KCl), and *ℓ* = 1.0 cm.

As a next step, the redox properties of the copper(II) complexes were investigated by cyclic voltammetry at pH 7.4 in a 90% DMSO/H_2_O mixture to provide sufficient solubility. The voltammograms revealed reversible processes (Figure 5a), and formal potentials could be calculated (Table 3 and Table S1). The potentials were in the −0.24 to −0.17 V range and were in the following order for the copper(II/I) redox couples: COTI-NMe_2_ < COTI-NMeCy < COTI-2 < COTI-NH_2_. This is in the range of the formal potential at a physiological pH reported for oxidized/reduced glutathione (GSSG/GSH) of −0.26 V [21]. The direct redox reaction of the copper(II) complexes COTI-NH_2_ and COTI-2 with GSH was already investigated spectrophotometrically in our previous work [16]. In both cases, mixed-ligand GSH-Cu(II)-TSC complexes were formed in an initial step. Then, a relatively slow redox reaction took place in the case of COTI-NH_2_, resulting in the formation of the Cu(I)-GSH complex and the free TSC ligand, similarly to how it was reported for the Triapine complex [15,30]. On the contrary, COTI-2 behaved differently, since, after the formation of the mixed-ligand adduct with GSH, the spectra were almost unchanged within 6 h, suggesting that GSH could not reduce this copper(II) complex under the used conditions. The redox-stable GSH-Cu(II)-COTI-2 complex was suggested to be the species that is recognized and transported by ABCC1 (see below) [16]. Therefore, the same experiment was performed for the copper(II) complexes of COTI-NMe_2_ (Figure 5b) and COTI-NMeCy in the presence of 300 equiv. GSH (Figure S16). For both compounds upon the addition of GSH, a small shift in the λ_max_ value around 400 nm could be observed, which could be explained by the formation of a mixed-ligand GSH adduct, but then no measurable spectral changes were seen. In contrast to the iron(III) complexes, the weaker reducing agent AA was not able to reduce any of the copper(II) complexes, which is in line with the lower redox potential of the latter (data not shown). The high stabilities of the ternary complexes of COTI-NMe_2_ and COTI-NMeCy suggest that they were also substrates for the ABCC1-mediated drug efflux and resistance.

Finally, zinc(II) complexation of COTI-2, COTI-NH_2_, COTI-NMe_2_, and COTI-NMeCy was studied by UV-vis spectrophotometry (representative spectra for the zinc(II)–COTI-NMeCy system are shown in Figure S17). Stability constants for [ZnLH]^2+^, [ZnL]^+^, and [ZnL_2_] were determined by the deconvolution of the spectra similarly as for iron and copper complexes (Table 3). As Zn complexes are not redox-active under physiological conditions, no investigations regarding reduction processes were performed. In good agreement with the literature on other p53 re-activating TSCs, such as ZMC2 [31,32], all COTI-2 derivatives showed a similar affinity for zinc, distinctly higher than Triapine. Consequently, the derivatization probably did not interfere with the p53-restoring potential of the new COTI-2 variants. Notably, although the zinc binding of the COTI-2 derivatives was stronger than that for Triapine, the data suggest that, at low nM drug concentrations, the zinc complexes were most likely dissociated. However, unlike copper(II), divalent zinc can form mono- as well as bis-complexes, which could also impact its interaction with the p53 molecule.

**Figure 6:**
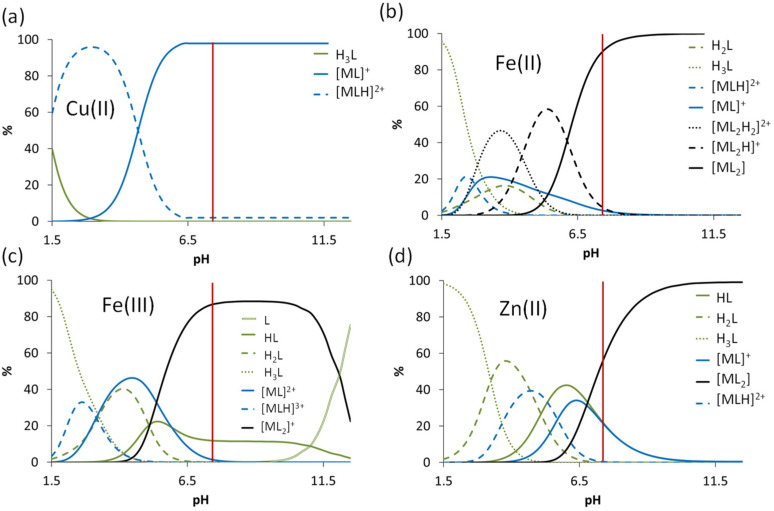
Calculated concentration distribution curves for (**a**) Cu(II):COTI-2 = 1:1, (**b**) Fe(II):COTI-2, (**c**) Fe(III):COTI-2 = 1:2, and (**d**) Zn(II):COTI-2 = 1:2 system (*c*_L_ = *c*_Cu(II)_ = 1 µM, *c*_Fe(II)_ = *c*_Fe(II)_ = *c*_Zn(II)_ = 0.5 µM, 30% (*v/v*) DMSO/H_2_O, *T* = 25 °C, and *I* = 0.10 M (KCl)). The red lines indicate pH 7.4.

A summary of the calculated metal-binding profile of 1 µM COTI-2 with 1:1 copper(II) and 2:1 iron(II), iron(III), and zinc(II) is depicted in Figure 6. These concentration distribution curves clearly show that COTI-2 was deprotonated at the hydrazinic NH in all complexes present at pH 7.4 under these conditions (except for a small fraction of [Fe(II)L_2_H]^+^). Furthermore, in the case of copper(II) and iron(II), COTI-2 was exclusively bound to the metal ions mainly as [Cu(II)L] and [Fe(II)L_2_], respectively. In the presence of iron(III), the major species was [Fe(III)L_2_]^+^, and for zinc(II), the complex [Zn(II)L_2_] was the major species, both with ~10–20% of free COTI-2 ligand. This nicely represents the lower stability of COTI-2 complexes with these two metal ions. Notably, the lower the assumed concentrations in these metal–COTI-2 systems, the more ligands dissociate, resulting in the conversion of 1:2 into 1:1 complexes and/or higher levels of free COTI-2.

Taken together, our data indicate that, besides zinc, COTI-2, as well as its derivatives, can strongly interact with several other metal ions, including iron and copper. In general, this observation is expected, as most TSCs show a broad metal interaction profile including the p53 re-activator ZMC1, where the chelation of iron and copper has also been reported [33]. However, no further investigations into the role of these metals in the biological activity of COTI-2 and other p53-restoring TSCs have been performed so far. This is noteworthy, as the affinity of all drugs for zinc was lower in comparison with those for copper and iron. Moreover, as even small changes in the chemical structure already distinctly impact the chemical characteristics of anticancer TSCs, we became interested in how these chemical differences influence the biological behavior of the drugs, especially with respect to drug resistance.

### 3.5. Anticancer Activity and Thiosemicarbazone Cross-Resistance Profiles of COTI-NMe_2_ and COTI-NMeCy

**Figure 7:**
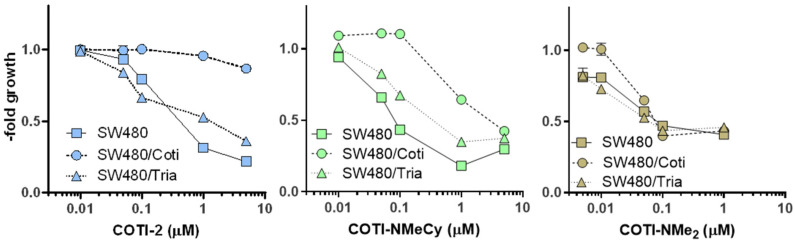
Fold viability plotted against the drug concentration to assay the resistance of SW480/Tria and SW480/Coti cells against COTI-NMeCy, COTI-NMe_2_, and COTI-2. The anticancer activity of the drugs was tested by MTT viability assay after 72 h of drug incubation in SW480 cells vs. the resistant sublines. The mean ± standard deviation (SD) was derived from triplicates of one representative experiment out of three.

In order to assess the impact of the *N*-dimethyl and *N*-methyl-cyclohexyl substitution on the antitumor activity, COTI-NMe_2_ and COTI-NMeCy were tested against SW480 colon adenocarcinoma cells and our recently established cell lines with acquired thiosemicarbazone resistance against COTI-2 (SW480/Coti [16]) or Triapine (SW480/Tria [26]). Briefly, in the case of SW480/Coti cells, the resistance is based on ABCC1 overexpression [16], while SW480/Tria cells are characterized by the overexpression of the ABCB1 efflux pump, loss of phosphodiesterase 4D (PDE4D) [34,35], and overexpression of RR (Figure S18). Drug activity was assessed by MTT assays performed after 72 h of incubation (Figure 7 and Table 4) and was determined in the presence of one equivalent of copper(II) or zinc(II) ions in selected cases. The results were compared with those of COTI-NH_2_, COTI-2, and Triapine, which were already analyzed in our previous publication [16].

It was already demonstrated in our previous publication that COTI-2 was slightly more active than Triapine in chemosensitive SW480 cells, while COTI-NH_2_ had a distinctly reduced anticancer efficacy [16]. This impact of terminal di-substitution is a known phenomenon, which has been already observed for several TSC subtypes [7,15,36,37]. With regard to the new derivatives, COTI-NMe_2_ was the most active, with an 11-fold lower IC_50_ value than COTI-2. Interestingly, the substitution of one terminal methyl group of COTI-NMe_2_ with a cyclohexane ring (COTI-NMeCy) reduced the activity back to the levels of the parental compound COTI-2. These differences between COTI-NMe_2_ and COTI-2 as well as COTI-NMeCy are surprising, as, in the case of the di-2-pyridyl (Dp) series, the N-Me_2_ (Dp44mT) and the N-chexyl (DpC) derivatives both show comparable IC_50_ values in the nanomolar range [38]. Additionally, considering the above-described physico-chemical properties, these results were rather unexpected. With regard to the impact of metal chelation, copper complexation enhanced the cytotoxicity of the TSCs, especially in the case of COTI-2 [16] and COTI-NMeCy (7–8 fold). In contrast, Zn chelation had no impact on the anticancer activity of COTI-2, which might be explained by the reduced stability of the complex at such low concentrations, in line with the lower pM value (compare Table 3).

With regard to the impact of TSC resistance, SW480/Tria cells (see Figure S18 for the confirmation of the Triapine resistance) were only cross-resistant to COTI-NH_2_, but not to COTI-2 itself or all other derivatives. As SW480/Tria cells are also characterized by RR overexpression, this might indicate that, in contrast to Triapine, RR is not an important target of COTI-2.

Our previous investigation revealed that COTI-2 (but not COTI-NH_2_) is an ABCC1 substrate, which is associated with the formation of the stable ternary adduct of the copper complex with GSH [16]. Based on the GSH interaction data described above (compare Figure 5), we expected that the new COTI-2 derivatives COTI-NMe_2_ and COTI-NMeCy also form copper–GSH adducts in the cell and, consequently, are also recognized by ABCC1. In line with these expectations, SW480/Coti cells were cross-resistant to COTI-NMeCy (6.2-fold), Cu-COTI-2 (9.2-fold), and Cu-COTI-NMeCy (3.6-fold), although the resistance was weaker compared with COTI-2 (18.9-fold). However, surprisingly COTI-NMe_2_ was highly active against SW480/Coti, resulting in widely similar activity in the low-nM range in both parental and resistant cells. Moreover, in contrast to COTI-NH_2_ [34,35], this did not result in a shift to cross resistance to SW480/Tria cells. Interestingly, in the case of Cu-COTI-NMe_2_, a ~3-fold resistance of SW480/Coti cells was found. However, this difference did not reach statistical significance. Finally, comparable to COTI-2, Zn-COTI-2 was also affected by the acquired resistance of SW480/Coti cells, supporting the hypothesis that the Zn complex is, in fact, not stable at such concentrations under physiological conditions.

This resistance pattern of the new COTI-2 derivatives could be also confirmed in a known ABCC1-overexpressing cell model (GLC-4/adr) vs. the chemo-naive parental GLC-4 cells (Table 5; Figure S19). Thus, COTI-NMeCy (like COTI-2) showed reduced activity in GLC-4/adr cells, while COTI-NH_2_ and Triapine were not affected by the overexpression of the efflux pump. In the case of COTI-NMe_2_, a minor cross-resistance of 2.5-fold was seen, which did not reach statistical significance. This was further supported by the co-culture experiments with the known ABCB1/ABCC1 inhibitors verapamil and CSA, which (in contrast to COTI-2 and COTI-NMeCy) had no relevant impact on the activity of COTI-NMe_2_ (Figure S20). Taken together, these data indicate that, in contrast to COTI-2 and COTI-NMeCy, ABCC1 expression has only a small impact on the efficiency of COTI-NMe_2_.

### 3.6. Mode of Action Studies on COTI-NMe_2_

**Figure 8:**
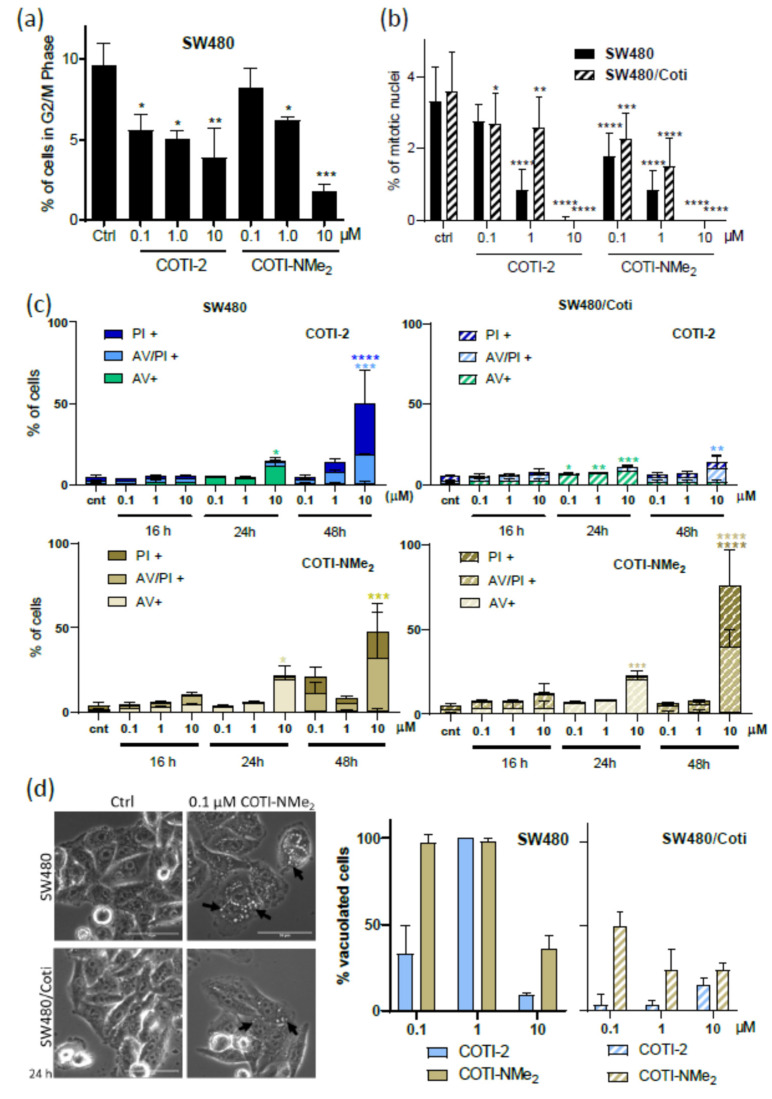
Cell cycle arrest and cell death induction by COTI-2 in SW480 and SW480/Coti cells after 24 h of treatment. (**a**) The percentage of cells in the G2/M phase was analyzed by staining ethanol-fixed cells with propidium iodide (PI) followed by flow cytometry after 24 h treatment. The mean ± SD was derived from three independent experiments. (**b**) Nucleic morphology was analyzed by the DAPI staining of methanol/acetone (1:1)-fixed cells after 24 h of treatment. Microscopic images were captured and mitotic cells were counted with ImageJ. The mean ± SD was derived from triplicates of three independent experiments. (**c**) Cell death induction was analyzed by annexin V (AV) and PI staining followed by flow cytometry. The mean ± SD was derived from three independent experiments after treatment at the indicated time points. (**d**) Microscopy images of paraptotic vesicle formation (indicated by black arrows) in SW480 and SW480/Coti cells after 24 h of treatment with COTI-2 and COTI-NMe_2_, and the percentage of vacuolated cells was counted with Image J. The mean ± SD was derived from three independent experiments. Significance was calculated in (**a**) with one-way ANOVA and the Holm-Sidak’s multiple comparisons test, (**b**) two-way ANOVA with Sidak’s multiple comparisons test and (**c**) two-way ANOVA with Dunnett’s multiple comparisons test (*p* < 0.05 *, *p* < 0.01 **, *p* < 0.001 ***, *p* < 0.0001 ****).

As the next step, we investigated whether there were differences in the mechanisms underlying the anticancer activity of COTI-2 and COTI-NMe_2_. It has been repeatedly shown that (probably due to their iron-chelating properties) TSCs have undoubtedly an impact on the proliferation and cell cycle distribution of cancer cells [4]. Accordingly, flow cytometry analysis of PI-stained, ethanol-fixed SW480 cells showed that both COTI-2 and COTI-NMe_2_ induced a significant reduction in the G2/M population (Figure 8a and Figure S21). Further analysis of the mitotic fraction alone (with DAPI stained nuclei) revealed a (complete) loss of the mitotic cell fraction for both drugs, with COTI-NMe_2_ being slightly more effective in the nanomolar concentration range (Figure 8b), which is in line with its distinctly lower IC_50_ value. In agreement with the resistance profiles observed in the viability assays, in the DAPI stains, SW480/Coti cells were also resistant to COTI-2, while there was no significant difference in the case of COTI-NMe_2_. Additionally, with regard to cell death induction, COTI-NMe_2_ was able to break the resistance of SW480/Coti cells, as shown by the AV/PI stains (Figure 8c). As described in the introduction section, we recently reported that COTI-2 is able to induce paraptosis, a quite recently described form of programmed cell death [16]. Thus, we were also interested in the paraptosis-induction potential of COTI-NMe_2_. One hallmark of paraptosis is the appearance of ER-derived cytoplasmic vesicles [39,40]. As shown in Figure 8d, COTI-NMe_2_ was more effective in this respect than COTI-2 in SW480 cells, with 100% of the cells presenting paraptotic features upon treatment with 0.1 µM and 1 µM. The percentage of cells with paraptotic vesicles again declined upon treatment with 10 µM of the drugs, which can be explained by the high apoptosis rates observed after 48 h (compare Figure 8c), indicating that apoptosis plays a more pronounced role at higher concentrations. Interestingly, when considering the impact of resistance on this aspect in the mode of action, the paraptosis-inducing potential was distinctly reduced for both drugs in SW480/Coti compared with SW480 cells, although the difference was less pronounced for COTI-NMe_2_ than for COTI-2.

**Figure 9:**
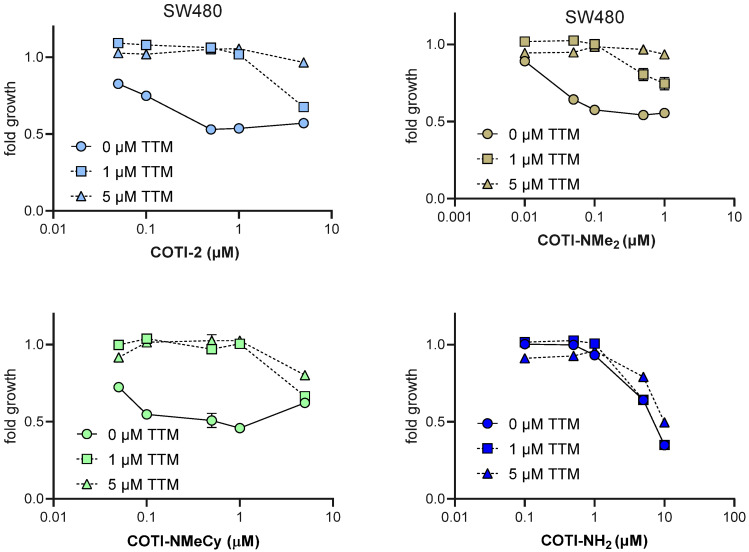
Impact of tetrathiomolybdate (TTM, 1 µM, and 5 µM) on the anticancer activity of COTI-2 and its derivatives in SW480 cells. Viability was measured by MTT assay after 72 h of combined drug treatment. The mean ± SD was derived from triplicates of one representative experiment out of three.

Finally, based on the strong affinity of the tested compounds for copper ions and their importance in terms of activity and resistance, we were interested in the impact of the known copper chelator TTM on the anticancer activity of COTI-2 and COTI-NMe_2_. For comparison, COTI-NMeCy and COTI-NH_2_ were also tested in these experiments. In agreement with data for Dp44mT [41], which propose that the copper complex is the active species, the addition of TTM exhibited strong protective activity against COTI-2, COTI-NMe_2_, and COTI-NMeCy, but not against COTI-NH_2_ (Figure 9). In general, this pattern is in line with the lower copper-binding affinity of COTI-NH_2_ (compare Figure 6) and the distinctly reduced stability of the respective ternary GSH-Cu(II)-thiosemicarbazone complex (compare Figure 5 and ref. [16]). Moreover, together, these data indicate that, despite being more active than COTI-2 and presenting a reduced impact of acquired COTI-2 resistance, the underlying mode of activity of COTI-NMe_2_ still seems to be widely similar to that of its parental compound. Consequently, we hypothesized that COTI-NMe_2_ efflux by SW480/Coti cells is less efficient than that of COTI-2.

### 3.7. Cellular Accumulation of COTI-2 and COTI-NMe_2_ in SW480 and SW480/Coti Cells

**Figure 10:**
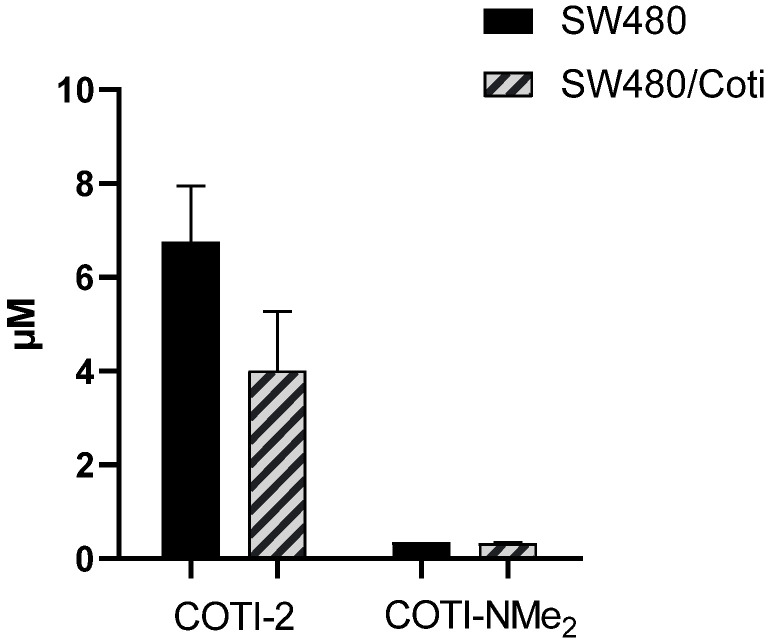
LC-MS measurements of the intracellular levels of free COTI-2 and COTI-NMe_2_ in SW480 and SW480/Coti cells incubated with a 10 µM drug concentration for 3 h.

ABCC1-mediated drug resistance is based on drug efflux, which results in lower intracellular drug levels and, thus, reduced target inhibition. Therefore, we further investigated the hypothesis that the differences in activity between COTI-2 and COTI-NMe_2_ in the resistant ABCC1-expressing SW480/Coti cells could be explained by the differences in cellular drug uptake. We evaluated the cellular drug levels after 3 h of incubation and extraction with acetonitrile via liquid chromatography–mass spectroscopy (LC-MS). As a first step, pre-experiments with the spiking of the drugs into SW480 extracts were performed, which showed good extractability under the used conditions (see the Experimental Part). When evaluating the drug levels in treated cells, in line with our expectations, the uptake of COTI-2 was ~40% lower in resistant SW480/Coti cells compared with the parental SW480 cells (Figure 10). In contrast, there was no difference in the cellular COTI-NMe_2_ levels between the two cell lines. Unexpectedly, despite its distinctly improved anticancer activity compared with COTI-2, for COTI-NMe_2_ markedly lower drug levels (10–20-fold) were detected inside both cell clones. The explanation of these differences is not trivial. One possibility could be the different protein binding behaviors or intracellular distributions of the compounds, which prevent/hamper the extraction of COTI-NMe_2_ by acetonitrile. This would also mask COTI-NMe_2_ from recognition by ABCC1 and, thus, explain why (despite its formation of stable copper complexes) it is only weakly affected by the overexpression of this efflux pump. Alternatively, COTI-2 might have so far unknown extracellular targets (e.g. scavenging of nutrients), to which COTI-NMe_2_ binds more efficiently. Further analytical investigation of the intra- and extracellular distribution and protein-binding pattern is a matter of future studies.

## 4. Conclusions

COTI-2 is a new thiosemicarbazone, which is currently being evaluated in a clinical phase I trial for the treatment of advanced gynecological cancers. With regard to its mode of action, COTI-2 has been considered as a re-activator of certain p53 mutations. In more detail, it is supposed to interact specifically with p53 variants in the zinc finger domain of the protein, resulting in the restoration of a “wild-type”-like conformation [11,12,13]. An interaction with endogenous metal ions is generally expected, as COTI-2 is a representative of the thiosemicarbazone compound class, well-known for its strong metal-chelating ability. Consequently, it is very surprising that the metal interaction profile of COTI-2 has not been investigated in-depth so far. Moreover, the impact of COTI-2 derivatization on chemical and biological properties is widely unexplored.

During recent years, we and others described that very small changes in the structure of TSCs can have a distinct impact not only on the anticancer activity, but also on the recognition by drug-resistance mechanisms [8,15,16,36]. This knowledge is especially important when considering that COTI-2 is clinically developed in cancer patients with advanced disease, which have already developed resistance to several other (chemo)therapeutics.

In this study, two novel *N*-disubstituted COTI-2 analogues (COTI-NMe_2_ and COTI-NMeCy) were synthesized, characterized, and compared with the non-substituted COTI-NH_2_ derivative and COTI-2 itself. As a starting point, the solution chemical properties and complexation with iron(II), iron(III), copper(II), and zinc(II) of COTI-2 and its derivatives were studied for the first time. Additionally, the influence of the substituents in the other derivatives was analyzed. All TSCs revealed efficient chelation abilities for iron(II) and iron(III). COTI-2 showed the highest preference for iron(II) and COTI-NMe_2_ showed the highest preference for iron(III) among the tested compounds. The iron(III)/iron(II) redox potentials were in the range of α-*N*-pyridyl-TSCs, such as Triapine, and the iron(III) complexes could be reduced by GSH or AA to iron(II) species. Additionally, subsequent re-oxidation was observed, suggesting the ability to undergo the redox cycle. Interestingly, although all COTI derivatives had a higher affinity for zinc than, for example, Triapine, their affinity for iron(II) and copper(II) still exceeded their preferences for zinc. This indicates that, for COTI-2, interactions with metal ions other than zinc might also contribute to their mode of action. Indeed, we found that the copper chelator TTM distinctly protected cells from the activity of COTI-2, COTI-NMe_2_, and COTI-NMeCy, suggesting a role of copper in their mode of action.

Our recent work indicated that the interaction with copper(II) ions and the stability of the forming complexes seemed to be crucial in the mode of action of certain TSCs, as it allowed the formation of ternary complexes with GSH and, in turn, recognition by the efflux pump ABCC1. All COTI-2 derivatives formed highly stable copper(II) complexes in the order of COTI-NH_2_ < COTI-NMe_2_ < COTI-NMeCy < COTI-2, and ternary complex formation with GSH was observed for all of them. However, reduction by this reducing agent only took place in the case of the complex with COTI-NH_2_, possessing the highest redox potential.

Based on the cytotoxicity assays performed on the ABCC1-overexpressing cancer cells, COTI-2 and COTI-NMeCy could be identified as ABCC1 substrates most probably based on the recognition and efflux of their GSH-copper(II)-TSC ternary species. In contrast, for COTI-NH_2_, the GSH adduct suffers from fast reduction and dissociation; therefore, it cannot be efficiently transported by ABCC1. Interestingly, although ABCC1 overexpression had only a minor impact on its anticancer activity, for COTI-NMe_2_, a stable copper(II)-TSC-GSH adduct could also be observed. One explanation of this effect could be that COTI-NMe_2_ had a distinctly lower IC_50_ value (~10-fold) than the other tested COTI-2 derivatives in SW480 cells. Consequently, at such low concentrations, the probability of forming copper complexes might be distinctly weakened, preventing the subsequent formation of stable GSH–conjugates. The reasons underlying this enhanced activity of COTI-NMe_2_ compared with COTI-2 in SW480 cells are currently widely unclear. Interestingly, all biological investigations indicated that the two drugs are quite similar in their mode of action (cell cycle arrest in the G1/S phase, induction of paraptosis at nanomolar concentrations, apoptotic cell death at higher drug concentrations, dependence on copper complex formation, etc.). The only distinction was observed in their cellular drug uptake, although the differences were counter-intuitive, as we found lower levels of “free” COTI-NMe_2_ inside the cells than of COTI-2. One theory that could explain this observation is that COTI-NMe_2_ might have a different intracellular protein-binding and distribution pattern, which results in more efficient binding or delivery to its target. Whether this is associated with p53 (SW480 has two different p53 mutations) or one of the multiple other targets that have been discussed for TSCs [4,42,43,44,45,46] needs to be further evaluated in future studies. Moreover, it will be interesting to also evaluate the activity of COTI-NMe_2_ in other cancer cell models in cell cultures, as well as in vivo. Taken together, COTI-NMe_2_ is an interesting candidate for subsequent biological investigations to further elucidate the reasons for the enhanced biological activity and altered resistance profile compared with COTI-2.

## Figures and Tables

**Table 1:** p*K_a_* values determined by UV-vis spectrophotometric titrations in 30% (*v/v*) DMSO/H_2_O (*I* = 0.1 M (KCl)); measured and predicted solubility (*S*), log*D*_7.4_ (*n*-octanol/water), and effective passive permeability (*P_eff_*) values at 25 °C.

	COTI-2	COTI-NH_2_	COTI-NMe_2_	COTI-NMeCy
p*K_a_* (H_3_L ^a^)	3.23 ± 0.01	*–*	*–*	*–*
p*K_a_* (H_2_L ^a^)	5.08 ± 0.01	4.39 ± 0.01	3.65 ± 0.01	3.47 ± 0.01
p*K_a_* (HL ^a^)	10.97 ± 0.01	12.22 ± 0.01	12.09 ± 0.01	12.40 ± 0.01
*S* (calc., μM) ^b^	5.6	954	324	31
*S* (meas., μM)	0.41	737	417	3.9
log *D*_7.4_ (calc.) ^c^	+2.99	+1.43	+1.88	+3.68
log *D*_7.4_ (meas.)	*–*	+1.64 ± 0.01	*–*	*–*
*P_eff_* (×10^−6^ cm s^−1^)	*–*	6.7 ± 0.7	16.0 ± 0.1	*–*

^a^ Charges are omitted for clarity. ^b^ Predicted by SwissADME (http://www.swissadme.ch, accessed on 20 August 2022). ^c^ Predicted by MarvinSketch software (MarvinSketch, version 16.12.12.0, calculation module developed by ChemAxon (Budapest, Hungary) http://www.chemaxon.com/products/marvin/marvinsketch/, accessed on 20 August 2022).

**Table 2:** Overall stability constants (log*β*) of the iron(III/II) complexes and formal redox potentials (*E*’) of the iron(III)–ligand (1:2) systems.

	COTI-2	COTI-NH_2_	COTI-NMe_2_	COTI-NMeCy
log*β* [Fe(III)LH]^3+^	21.16 ± 0.03	–	–	–
log*β* [Fe(III)L]^2+^	18.02 ± 0.03	20.00 ± 0.09	17.25 ± 0.02	17.06 ± 0.02
log*β* [Fe(III)L_2_]^+^	29.98 ± 0.06	32.29 ± 0.08	32.77 ± 0.03	32.20 ± 0.03
log*β* [Fe(II)LH]^2+^	21.00 ± 0.03	–	–	–
log*β* [Fe(II)L]^+^	18.28 ± 0.06	17.36 ± 0.06	17.62 ± 0.03	18.66 ± 0.03
log*β* [Fe(II)L_2_H_2_]^2+^	41.18 ± 0.06	–	–	–
log*β* [Fe(II)L_2_H]^+^	36.74 ± 0.09	–	–	–
log*β* [Fe(II)L_2_]	30.63 ± 0.09	31.21 ± 0.09	31.33 ± 0.09	31.04 ± 0.03
*pFe(III)*	18.6	18.5	19.2	18.0
pFe(III)*	10.6	10.4	11.1	9.9
*pFe(II)*	19.3	17.4	17.8	16.9
*E’* vs. NHE (V)	+0.11	+0.06	+0.06	+0.05

Conditions for the UV-vis spectrophotometric titrations: *T* = 25.0 °C, *I* = 0.10 M (KCl), 30% (*v/v*) DMSO/H_2_O}, pM (= –log[free metal ion as Fe(II) or Fe(III)]) values calculated at *c*_Fe_ = 1 μM, *c*_L_ = 10 μM, and pH = 7.40. pFe(III)*: Formation of the various Fe(III) hydroxido species as unbound metal was also considered. Conditions for cyclic voltammetry measurements: *c*_L_ = 1 mM, pH = 7.40 (10 mM HEPES), *T* = 25 °C, *I* = 0.1 M tetrabutylammonium nitrate, 90% (*v/v*) DMSO/H_2_O, and 0.01 V/s scan rate.

**Table 3:** p*K*_a_ values and conditional and overall stability constants (log*β_5.9′_* and log*β*) for the copper(II) and zinc(II) complexes, and formal redox potentials (*E*′) for the copper(II)–ligand (1:1) systems.

	COTI-2	COTI-NH_2_	COTI-NMe_2_	COTI-NMeCy
p*K*_a_[CuLH]^2+^	4.66 ± 0.01	2.24 ± 0.03	1.40 ± 0.02	1.26 ± 0.01
p*K*_a_[CuL]^+^	–	8.84 ± 0.04	>8	>8
log*β_5.9′_* [CuL]^+ a^	13.06 ± 0.02	10.59 ± 0.08	12.23 ± 0.05	12.56 ± 0.09
log*β* [CuL]^+ b^	18.19	16.92	18.42	19.08
*pCu*	15.6	13.1	14.7	15.0
*E*′ vs. NHE (V)	−0.19	−0.17	−0.24	−0.22
log*β* [ZnLH]^2+^	18.54 ± 0.02	–	–	–
log*β* [ZnL]^+^	12.85 ± 0.02	14.07 ± 0.04	13.02 ± 0.04	12.94 ± 0.04
log*β* [ZnL_2_]	23.23 ± 0.08	25.42 ± 0.04	25.24 ± 0.08	26.32 ± 0.09
*pZn*	11.9	11.6	11.7	12.1

Conditions for the UV-vis spectrophotometric titrations: *T* = 25.0 °C, *I* = 0.10 M (KCl), and 30% (*v/v*) DMSO/H_2_O. pZn and pCu values calculated at *c*_M_= 1 μM, *c*_L_ = 10 μM, and pH = 7.40. Conditions for cyclic voltammetry measurements: *c*_L_ = *c*_Cu(II)_ = 1 mM, pH = 7.40 (10 mM HEPES), *T* = 25 °C, *I* = 0.1 M tetrabutylammonium nitrate, 90% (*v/v*) DMSO/H_2_O, and 0.01 V/s scan rate. ^a^ Determined by an EDTA competition study by UV-vis spectrophotometry at pH = 5.90. log*β*’ for the [CuL]^+^ complex of Triapine = 9.47 calculated on the basis of the stability constants reported in [19]. ^b^ *β* [CuL]^+^ = *β*’ [CuL]^+^ × *α*_H_, where *α*_H_ = 1 + [H^+^]/*K*_a_ (HL) + [H^+^]^2^/(*K*_a_(HL) × *K*_a_(H_2_L^+^)); [H^+^] = 10^–5.90^ M.

**Table 4:** Anticancer activity (IC_50_ values after 72 h) of the indicated TSCs in SW480 cells, as well as the TSC-resistant SW480/Coti and SW480/Tria subclones.

Compound	SW480 (IC_50_, µM) ^a^	SW480/Coti (IC_50_, µM) ^a^	Relative Resistance ^b^	SW480/Tria (IC_50_, µM) ^a^	Relative Resistance ^b^
COTI-2	0.37 ± 0.17	9.51 ± 3.11^c^	25.7 ***	0.41 ± 0.16 ^c^	1.1 ^n.s.^
COTI-NMe_2_	0.04 ± 0.01	0.05 ± 0.03	1.3 ^n.s.^	0.04 ± 0.02	1.0 ^n.s.^
COTI-NMeCy	0.64 ± 0.10	3.98 ± 0.40	6.2 **	0.71 ± 0.17	1.1 ^n.s.^
COTI-NH_2_ ^c^	7.85 ± 0.92	4.39 ± 0.56	0.6 ***	>20	>2.5
Cu-COTI-2	0.05 ± 0.02	0.46 ± 0.09	9.2 **	n.d.	-
Cu-COTI-NMe_2_	0.01 ± 0.01	0.03 ± 0.04	3.0 ^n.s.^	n.d.	-
Cu-COTI-NMeCy	0.080 ± 0.003	0.31 ± 0.03	3.9 **	n.d.	-
Zn-COTI-2	0.420 ± 0.009	>10	>23.4	n.d.	
Triapine ^c^	0.82 ± 0.18	0.81 ± 0.08	1.0 ^n.s.^	>50	>61

^a^ IC_50_ values were calculated from concentration–response curves. Values are given as the mean ± SD of three independent experiments performed in triplicate. ^b^ Differences in sensitivity calculated by dividing the IC_50_ values of the resistant subline by those of the parental line. *** *p* ≤ 0.001, ** *p* ≤ 0.01; n.s. (not significantly different), calculated by a one-sample *t*-test. ^c^ Taken from [16].

**Table 5:** Anticancer activity (IC_50_ values after 72 h) of the indicated TSCs in GLC-4, as well as its ABCC1-overexpressing subclone GLC-4/adr.

Compound	GLC-4 (IC_50_, µM) ^a^	GLC-4/adr (IC_50_, µM) ^a^	Relative Resistance ^b^
COTI-2 ^c^	0.03 ± 0.01	0.48 ± 0.15	17.3 *
COTI-NMe_2_	0.03 ± 0.03	0.06 ± 0.03	2.0 ^n.s.^
COTI-NMeCy	0.17 ± 0.10	0.54 ± 0.15	7.8 *
COTI-NH_2_ ^c^	3.64 ± 0.39	3.65 ± 0.41	1.0 ^n.s.^
Triapine ^c^	0.65 ± 0.09	0.57 ± 0.05	0.9 ^n.s.^

^a^ IC_50_ values were calculated from concentration–response curves measured by MTT assay. Values are given as the mean ± SD of two or three independent experiments conducted in triplicate. ^b^ Relative resistance was calculated by dividing the IC_50_ values of the resistant GLC-4/adr by those of the parental GLC-4 cells. * *p* ≤ 0.05; n.s., not significantly different; calculated by one-sample *t*-test. ^c^ Taken from [16].

## Data Availability

The data presented in this study are contained within the article and the Supplementary Materials.

## Supplementary Materials

The following are available online at https://www.mdpi.com/article/10.3390/cancers14184455/s1. Crystallographic data for the crystal structure of [Cu(COTI-NH_2_)Cl_2_]·CH_3_OH were deposited with the Cambridge Crystallographic Data Centre, CCDC 2132417 [47,48,49,50,51,52,53,54]. Scheme S1:COTI-NMe_2_ NMR numbering scheme, Scheme S2: COTI-NMeCy NMR numbering scheme, Scheme S3: Synthetic route for COTI-NMeCy, Figure S1: ^1^H NMR spectrum of COTI-NMe_2_, Figure S2: ^13^C spectrum of COTI-NMe_2_, Figure S3: ^1^H NMR spectrum of COTI-NMeCy, Figure S4: ^13^C NMR spectrum of COTI-NMeCy, Figure S5: The anodic and cathodic peak current values for the (a) iron– COTI-NMeCy and (b) copper–COTI-NMeCy complexes plotted against the square root of the applied scan rates, Figure S6: UV-vis spectra and calculated individual molar absorption spectra of (a,b) COTI-NH_2_, (c,d) COTI-NMe_2_, (e,f) COTI-2 recorded at various pH values, Figure S7: Calculated concentration distribution curves for (a) COTI-2, (b) COTI-NH_2_, (c) COTI-NMe_2_, (d) COTI-NMeCy, Figure S8: UV-vis spectra of (a) COTI-NH_2_ and (b) COTI-Me_2_ recorded for the various phases in the PAMPA experiment, Figure S9: Concentration distribution curves calculated for the (a) iron(III)–COTI-2 (1:2) and (b) iron(III)–COTI-NMe_2_ (1:2) systems based on the overall stability constants, Figure S10: UV-vis spectra of the copper(II)–COTI-NH_2_–EDTA (1:1:x) system (x = 0–50) recorded after 3 h equilibration time, Figure S11: Experimental and simulated frozen solution EPR spectra recorded for the (a) copper(II)–COTI-2 and (c) copper(II)–COTI-NH_2_ systems. Calculated EPR spectra obtained for the various complexes of (b) COTI-2 and (d) COTI-NH_2_, Figure S12: ORTEP view of [Cu(COTI-NH_2_)Cl_2_]·CH_3_OH, Figure S13: Unit cell containing four molecules of [Cu(COTI-NH_2_)Cl_2_]·CH_3_OH, Figure S14: Packing arrangements in the crystal of [Cu(COTI-NH_2_)Cl_2_]·CH_3_OH, Figure S15: Packing arrangements in the crystal of [Cu(COTI-NH_2_)Cl_2_]·CH_3_OH showing some selected hydrogen bonds and π…π interactions, Figure S16: Time-dependent changes of the UV-vis absorption spectra of copper(II)– COTI-NMeCy (1:1) system in the presence of 300 equiv. GSH at pH 7.4, Figure S17: (a) UV-vis spectra of the zinc(II)– COTI-NMeCy (1:1) system recorded at various pH values, and (b) calculated individual molar absorption spectra of complex species, Figure S18: (a) Triapine resistance in SW480/Tria cells. (b) Protein expression of R1 and R2 investigated by Western blotting, Figure S19: Differences in viability (measured by MTT viability assay) of GLC-4 and ABCC1-overexpressing GLC-4/adr cells treated with COTI-2, COTI-derivatives and Triapine for 72 h, Figure S20: Impact of ABC transporter modulators on the anticancer activity of vincristine, COTI-2 and its derivatives in the SW480/Coti cells in comparison to the parental cells, Figure S21: Cell-cycle analysis by PI staining and flow cytometry of SW480 (A;B,C) cells, Table S1: Electrochemical data (cathodic and anodic peak potentials vs. Ag/AgCl/3 M KCl), peak separation, formal redox potentials vs. (NHE) for the copper(II)–ligand (1:1) and iron(III)–ligand (1:2) systems, Table S2: Anisotropic EPR spectroscopic parameters obtained for the studied copper(II) complexes of COTI-NH_2_ and COTI-2 in 30% (*v*/*v*) DMSO/H_2_O, Table S3: Isotropic EPR parameters calculated for the copper(II) complexes of COTI-NH_2_ and COTI-2 from the anisotropic data as an average of the g and A values (Table 5) and experimentally determined data for the copper(II)-complexes of Triapine, Table S4: Selected bond lengths (Å) and angles (°) for the complexes in crystal [Cu(COTI-NH_2_)Cl_2_]·CH_3_OH, Table S5: Hydrogen-bond geometry of in crystal [Cu(COTI-NH_2_)Cl_2_]·CH_3_OH, Table S6: Crystal data and structure refinement for [Cu(COTI-NH_2_)Cl_2_]·CH_3_OH.
