# Supplementary material for: Thiosemicarbazone Derivatives Developed to Overcome COTI-2 Resistance
Source: Cancers (Basel). 2022 Sep 14;14(18):4455. doi: 10.3390/cancers14184455 (PMC9497102; doi:10.3390/cancers14184455)
Supplement: Supplementary file 1 [file cancers-14-04455-s001.zip › cancers-1906787-supplementary.pdf]

SUPPORTING INFORMATION

Thiosemicarbazone derivatives developed to overcome COTI-2 resistance

Vivien Pósa, Alessia Stefanelli, Julia H. Bormio Nunes, Sonja Hager, Marlene Mathuber, Nóra V. May,
Walter Berger, Bernhard K. Keppler, Christian R. Kowol, Éva A. Enyedy, Petra Heffeter

Supplementary material and methods.

EPR spectroscopic studies on the copper(II) complexes of COTI-2 and COTI-NH_2

The experimental and simulated EPR spectra recorded at 77 K for the copper(II)–COTI-2 and copper(II)–COTI-NH_2 systems are shown in Figure S5 The anisotropic EPR spectroscopic parameters obtained for the studied copper(II) complexes are collected in Table S1, and the calculated isotropic EPR parameters are seen in Table S2. In all the three complexes ($[CuLH]^{2+}$, $[CuL]^{+}$, [CuL(OH)]) the nitrogen superhyperfine splitting is well resolved in the perpendicular region of the spectra (3200–3400 G), which could be simulated by taking into account two nitrogen donor atoms coordinating to the copper(II) ion. The nitrogen couplings were treated with rhombic parameters, from which one bigger value was obtained reflecting that one nitrogen is coordinated in the x and another from y directions referred to the *g*-tensor orientations. The deprotonation of $[CuLH]^{2+}$ caused an increase in the A_z hyperfine coupling and decrease in g_z value which shows and increasing ligand field around the metal ion. The formation of the mixed hydroxido [CuL(OH)] complex further increased the ligand field (g_z decreased and A_z increased), however, the A_y value significantly decreased comparing to the coupling of $[CuL]^{+}$.

Two series of pH-dependent EPR spectra were recorded: one at 0.08 mM Cu(II) and ligand concentration, and another one at 0.04 mM Cu(II) and 0.08 mM ligand concentrations in 30% (v/v) DMSO/water solution. At each pH value 0.30 mL samples were transferred into EPR tubes and the spectra were recorded in a dewar containing liquid nitrogen (77 K). The spectra were simulated by the “EPR” program [1] using rhombic g-tensor (g_x, g_y, g_z) and copper hyperfine tensor (A_x^{Cu}, A_y^{Cu}, A_z^{Cu}) parameters. The nitrogen super-hyperfine structure was taking into account with a rhombic hyperfine tensor (a_x^N, a_y^N, a_z^N) were the xyz directions referred to the g-tensor orientations. Orientation dependent linewidth parameters (α, β, and γ) were used to fit the linewidths through the equation $\sigma_{MI}=\alpha+\beta_{MI}+\gamma_{MI}^2$, where M_I denotes the magnetic quantum number of Cu(II) ions. Since a natural $CuCl_2$ was used for the measurements, the spectra were calculated by the summation of spectra ^{63}Cu and ^{65}Cu weighted by their natural abundances. The hyperfine and super-hyperfine coupling constants and the relaxation parameters were obtained in field units (Gauss = 10^{-4} T).

Structure of [Cu(COTI-NH_2)Cl_2]·CH_3OH complex determined by X-ray crystrallography

The structure of [Cu(COTI-NH_2)Cl_2]·CH_3OH is depicted in Figure S12. The complex crystallized in the monoclinic crystal system in $P2_1/c$ space group containing one complex and one methanol solvent molecule in the asymmetric unit. Selected bond distances and angles are collected in Table S4. The Cu(II) ion has a square pyramidal geometry with the coordination of {N,N,S} donor set of the ligand,

and one chloride ion (Cl1) coordinates equatorially and another chloride ion (Cl2) is found at the axial position. The Cu-Cl2 bond distance is significantly longer than that of Cu-Cl1. The cyclohexene ring of the dihydroquinoline moiety occupies two disordered positions where C6A occupies 73% and C6B 27%. The complex molecules arranged in a way that the equatorial coordination sphere is parallel to each other, and close π…π stacking interaction (3.7732(12) Å) could be measured between neighboring aromatic rings. The unit cell contains four molecules (compare Figure S13), and packing arrangements are depicted in Figures S14 and S15. Hydrogen bond geometry and crystal data can be found in Table S5 and S6.

X-ray crystrallography

Single crystals for the Cu(II) complex of COTI-NH_2 were obtained by in situ complex formation of the metal salt ($CuCl_2$) dissolved in water (c_{stock} = 0.1020 M) and the ligand (1.38 mg, 6.25×10^{-3} mmol) dissolved in methanol (1 ml), and the reactants were mixed in 1:1 ratio ($n_{ligand} = n_{Cu(II)} = 6.25\times10^{-3}$ mmol). X-ray diffraction data of crystal [Cu(II)(COTI-NH_2)Cl_2]$\cdot CH_3OH$ were collected at 295(2) K on a Rigaku RAXIS-RAPID II diffractometer using Mo-Kα radiation. Numerical absorption correction [2] was carried out using the program CrystalClear [3]. SHELX [4] program package under WinGX [5] software were used for structure solution and refinement. The structure was solved by direct methods. The models were refined by full-matrix least squares on F^2. Refinement of non-hydrogen atoms was carried out with anisotropic temperature factors. Hydrogen atoms were placed into geometric positions. They were included in structure factor calculations but they were not refined. The isotropic displacement parameters of the hydrogen atoms were approximated from the U(eq) value of the atom they were bonded to. The summary of data collection and refinement parameters is collected in Table S6. Selected bond lengths and angles of compounds were calculated by PLATON software [6]. The graphical representation and the edition of CIF files were done by Mercury [7] and EnCifer [8] softwares. The crystallographic data files for the complex have been deposited with the Cambridge Crystallographic Database as CCDC 2132417.

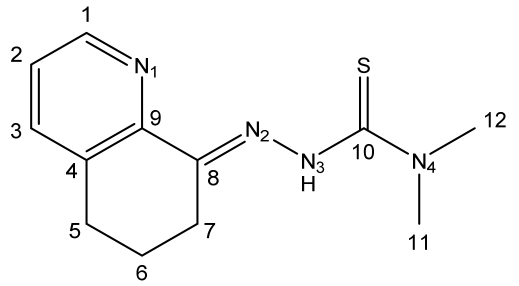

Scheme S1. COTI-NMe_2 NMR numbering scheme.

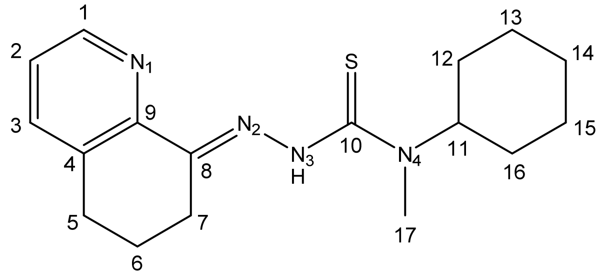

Scheme S2. COTI-NMeCy NMR numbering scheme.

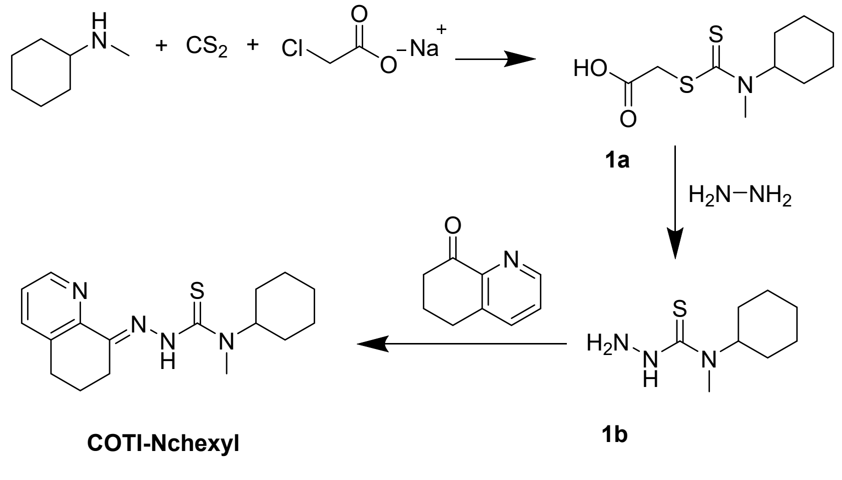

Scheme S3. Synthetic route for COTI-NMeCy.

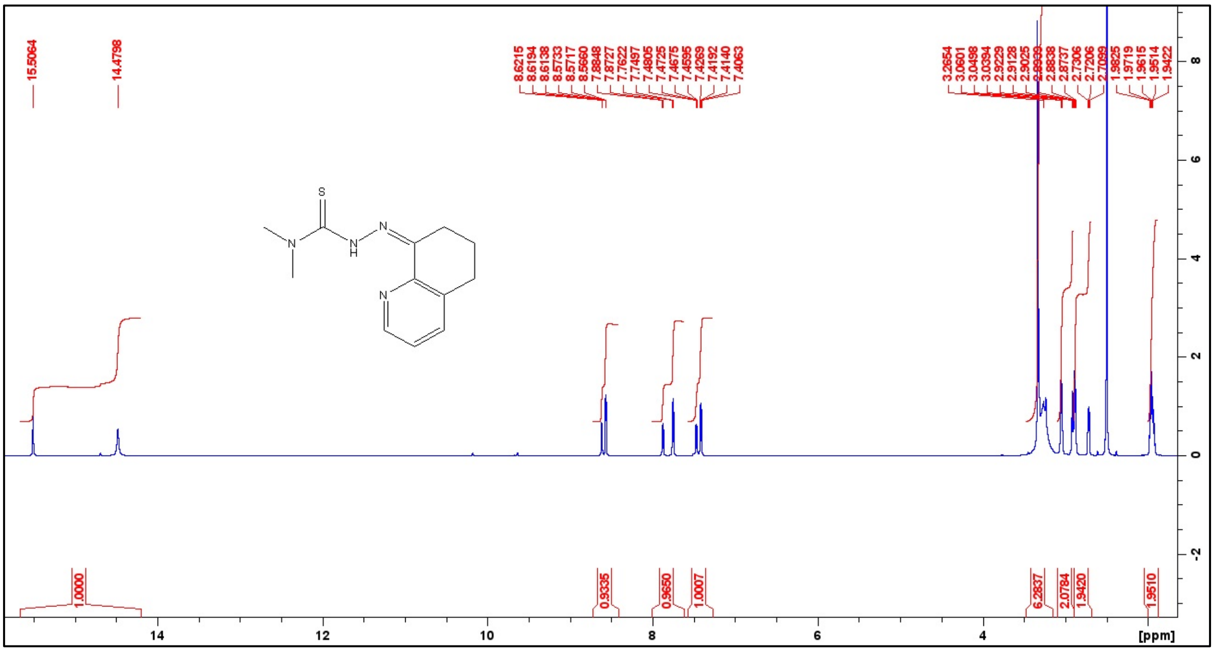

Figure S1. ^{1}H NMR spectrum of COTI-NMe_2.

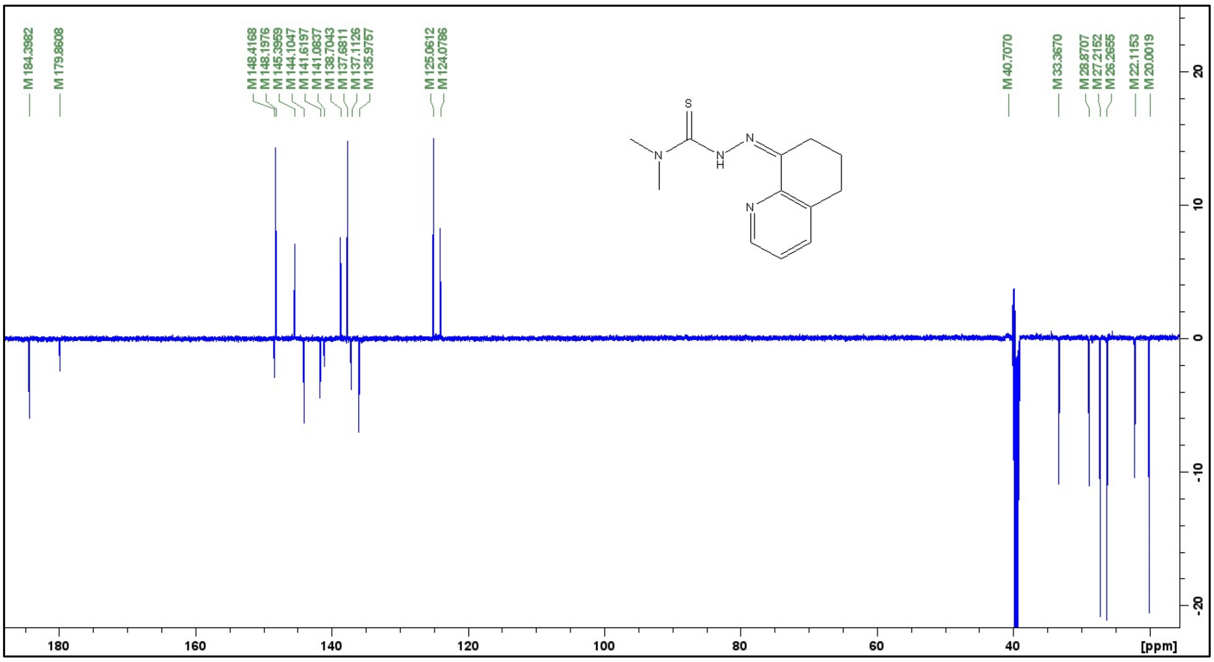

Figure S2. ^{13}C spectrum of COTI-NMe_2.

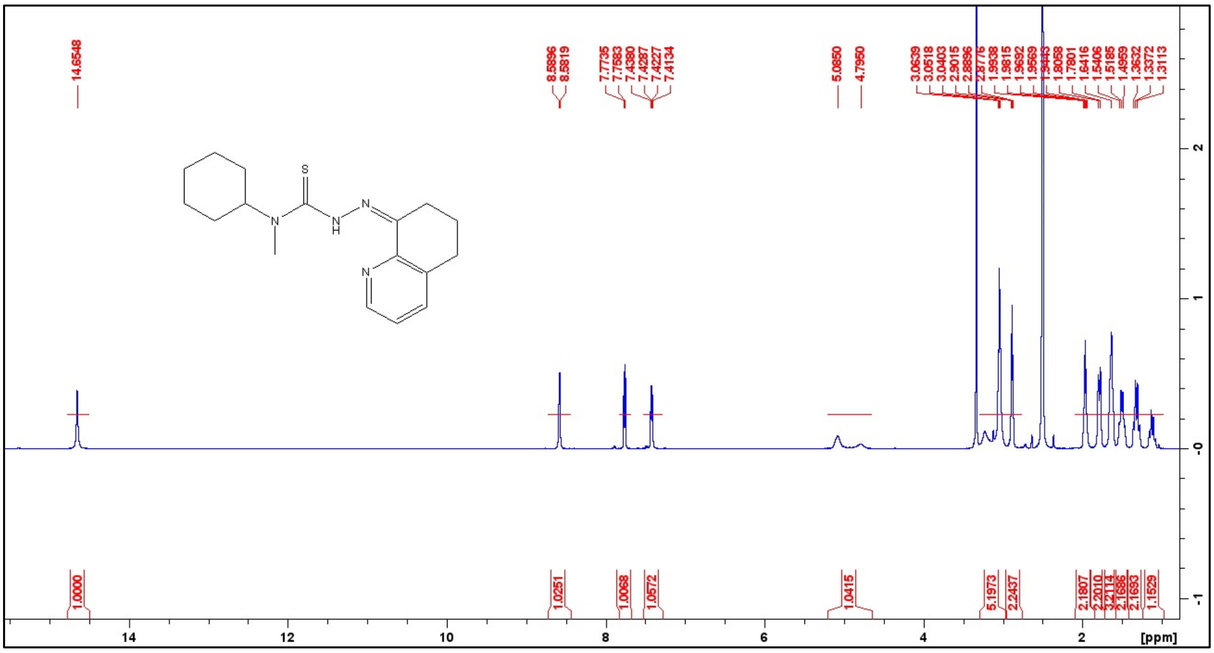

Figure S3. ^{1}H NMR spectrum of COTI-NMeCy.

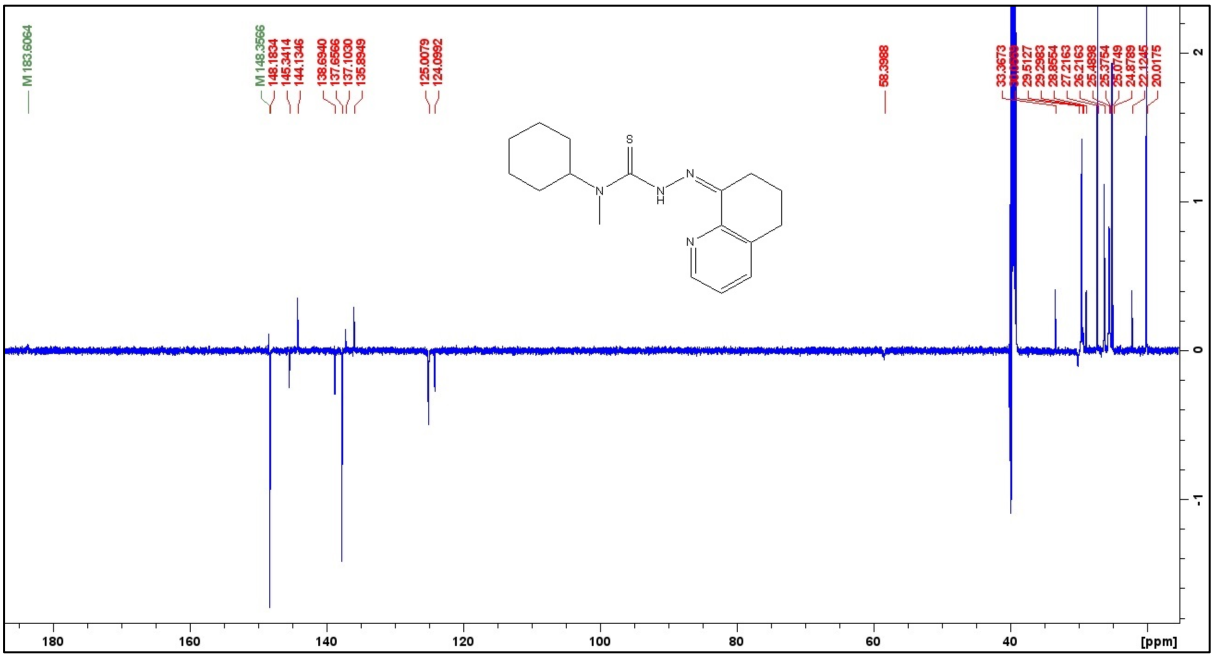

Figure S4. ^{13}C NMR spectrum of COTI-NMeCy.

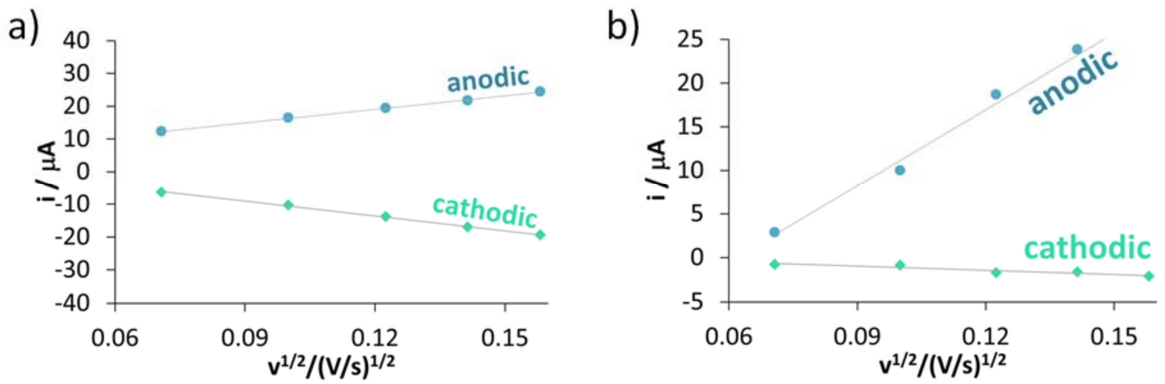

Figure S5. The anodic and cathodic peak current values for the a) iron– COTI-NMeCy and b) copper–COTI-NMeCy complexes plotted against the square root of the applied scan rates [c_L =1 mM; c_{Fe} = 0.5 mM; c_u = 1 mM; pH = 7.40 (10 mM HEPES); T = 25 ˚C; I = 0.1 M TBAN; 90% (v/v) DMSO/H_2O]. In the case of iron complexes, both curves are linear with similar slopes, which suggests diffusion controlled electrode reaction for the complexes in both oxidation states. Additionally, similar diffusion coefficients are likely due to the similar size and geometry of the iron(II) and iron(III) species. For the copper complexes, the curves show linearity suggesting that the redox species are freely diffusing in solution. The slopes for the cathodic and anodic currents differ, most likely the diffusion flux, thus the diffusion coefficient, is different for the copper(I) and copper(II) complexes.

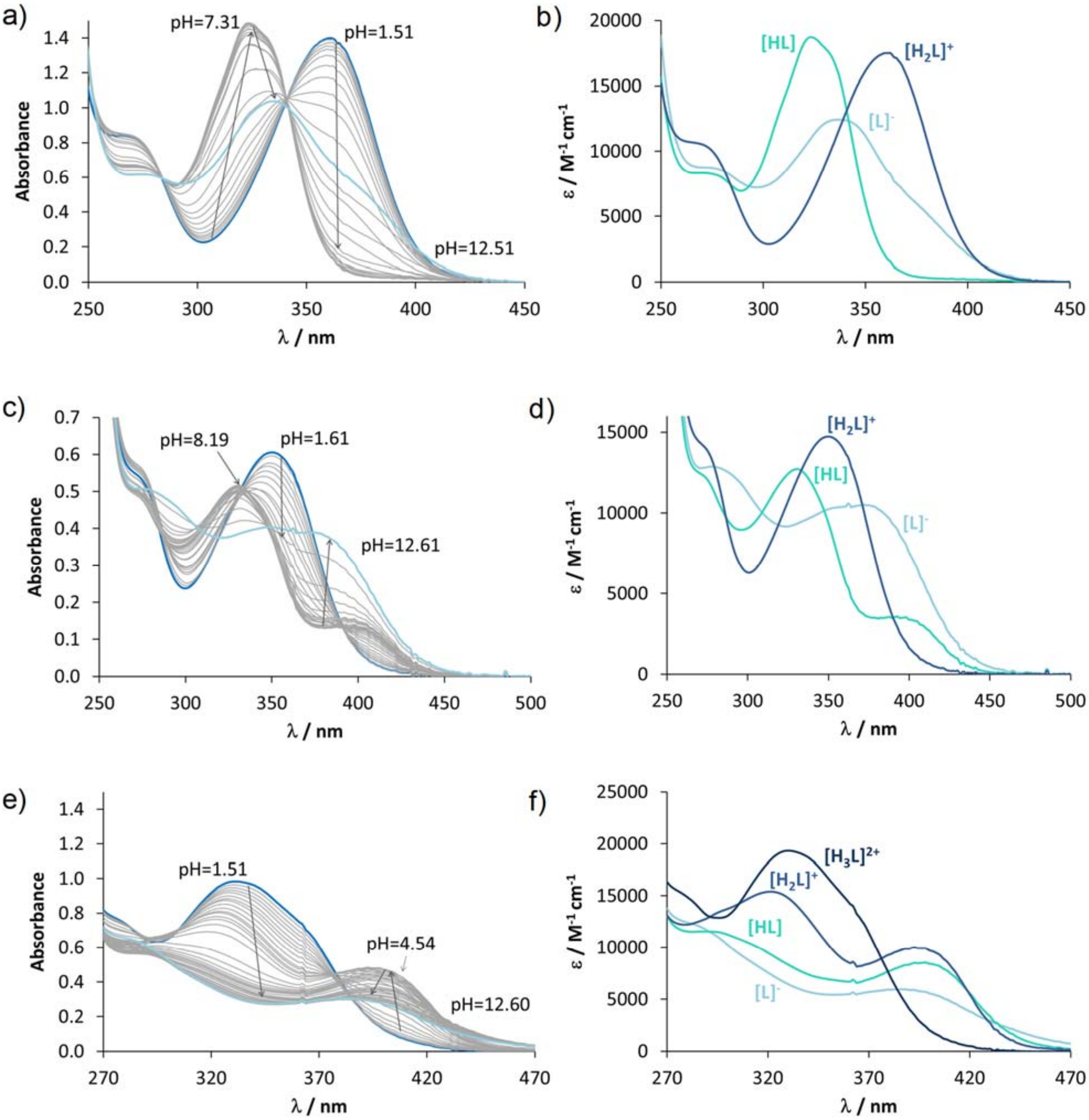

Figure S6. UV-vis spectra and calculated individual molar absorption spectra of a,b) COTI-NH_2, c,d) COTI-NMe_2, e,f) COTI-2 recorded at various pH values. *$c_{COTI\text{-}NH2}$ = 80 μM; $c_{COTI\text{-}NMe2}$ = 20 μM; $c_{COTI\text{-}2}$ = 10 μM; pH = 1.5 – 12.5; T = 25 ˚C; I = 0.10 M (KCl); ℓ = 2.0 cm (5 cm for COTI-2); 30% (v/v) DMSO/H_2O.*

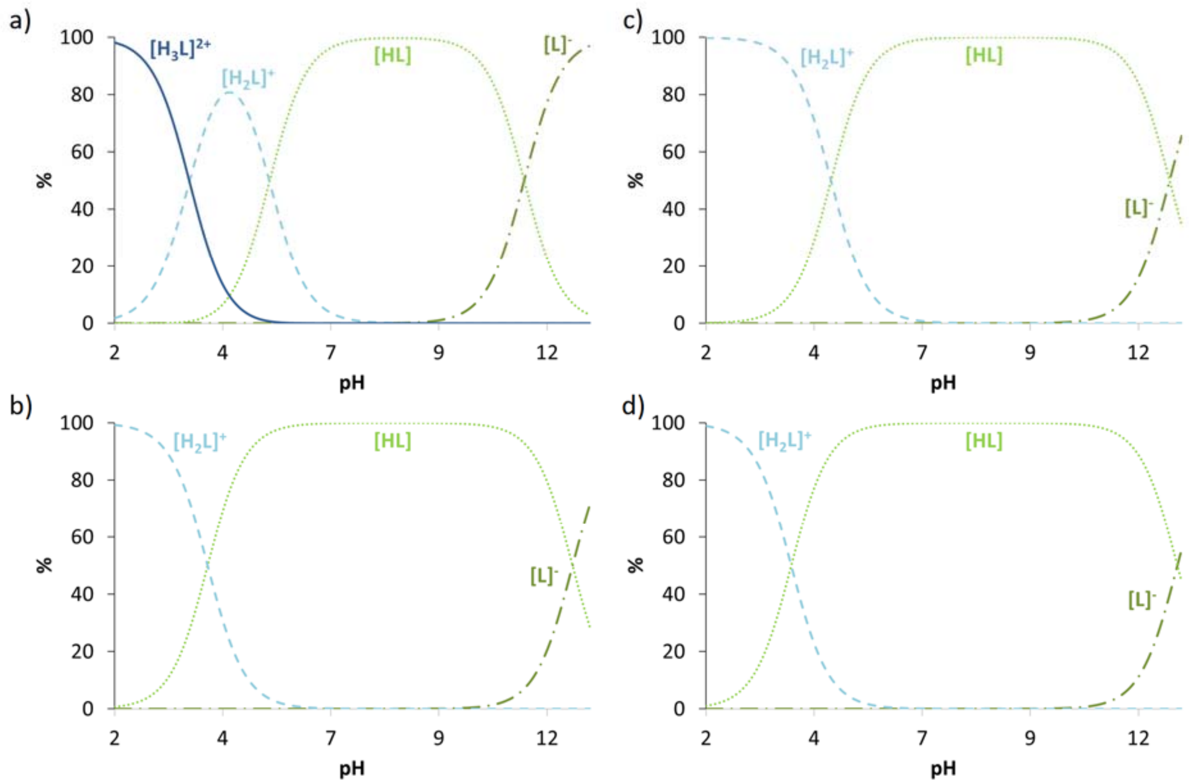

Figure S7. Calculated concentration distribution curves for a) COTI-2, b) COTI-NH_2, c) COTI-NMe_2, d) COTI-NMeCy. *{c_{ligand}= 20 µM; pH = 1.5 – 12.5; T = 25 ˚C; I = 0.10 M (KCl); 30% (v/v) DMSO/H_2O}*

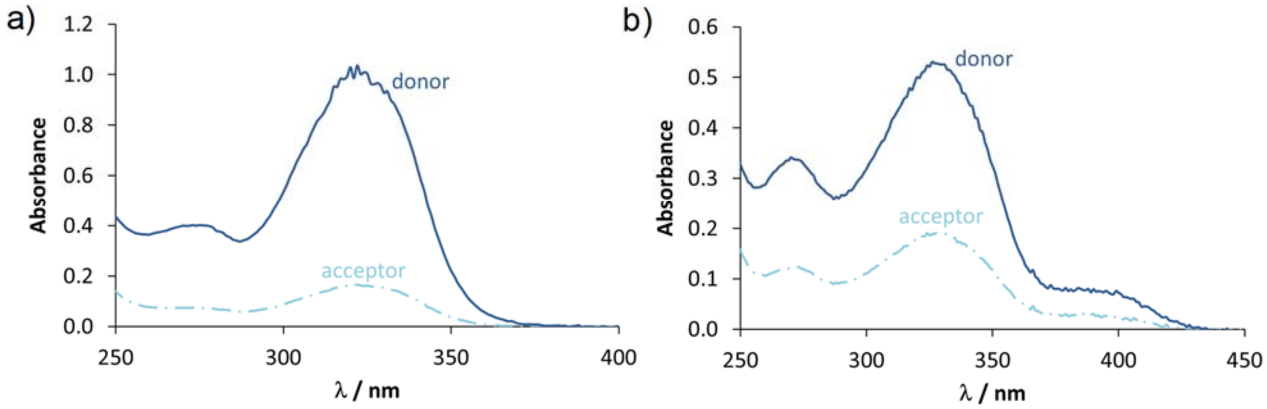

Figure S8. UV-vis spectra of a) COTI-NH_2 and b) COTI-Me_2 recorded for the various phases in the PAMPA experiment: donor phase (solid line), acceptor phase (dashed line). *$c_{compound}$ = 50 µM; pH = 7.40; T = 25˚C; I = 0.10 M (KCl); ℓ = 1.0 cm; c_{HEPES} = 15 mM.*

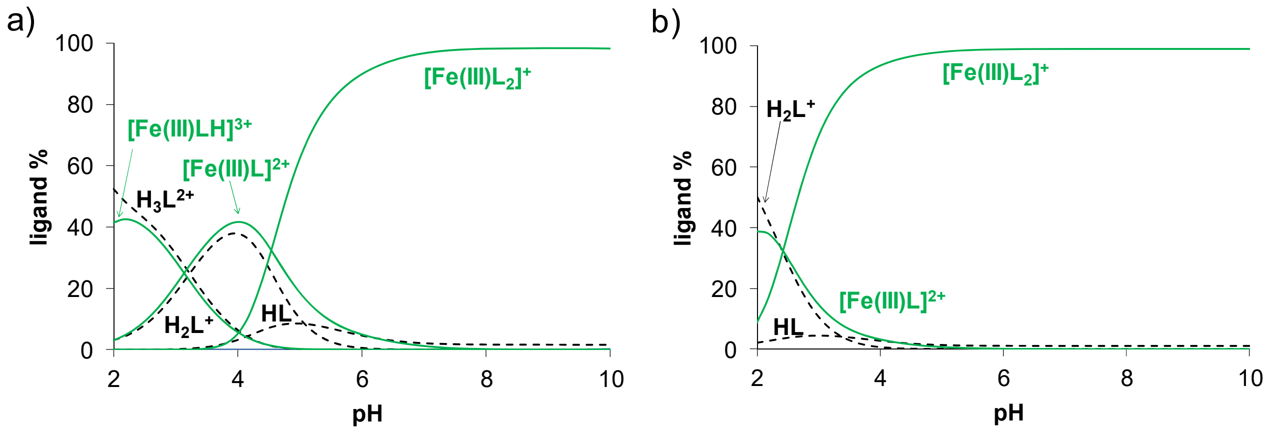

Figure S9. Concentration distribution curves calculated for the a) iron(III)–COTI-2 (1:2) and b) iron(III)–COTI-NMe_2 (1:2) systems based on the overall stability constants. $c_{Fe(III)}$ = 10 µM; c_{ligand} = 20 µM; T = 25˚C; I = 0.1 M (KCl).

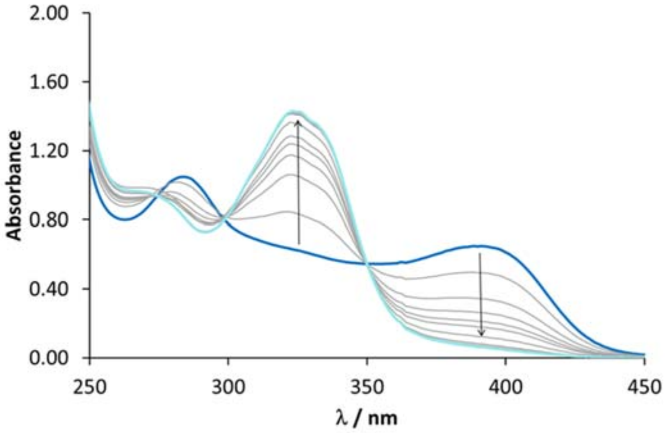

Figure S10. UV-vis spectra of the copper(II)–COTI-NH_2–EDTA (1:1:x) system (solid lines, x = 0–50) recorded after 3 h equilibration time. *$c_{Cu(II)}$ = c_L = 80 µM; c_{EDTA} = 0 – 4 mM; pH 5.9 (20 mM MES); T = 25 ˚C; I = 0.10 M (KCl); ℓ = 1.0 cm; 30% (v/v) DMSO/H_2O.*

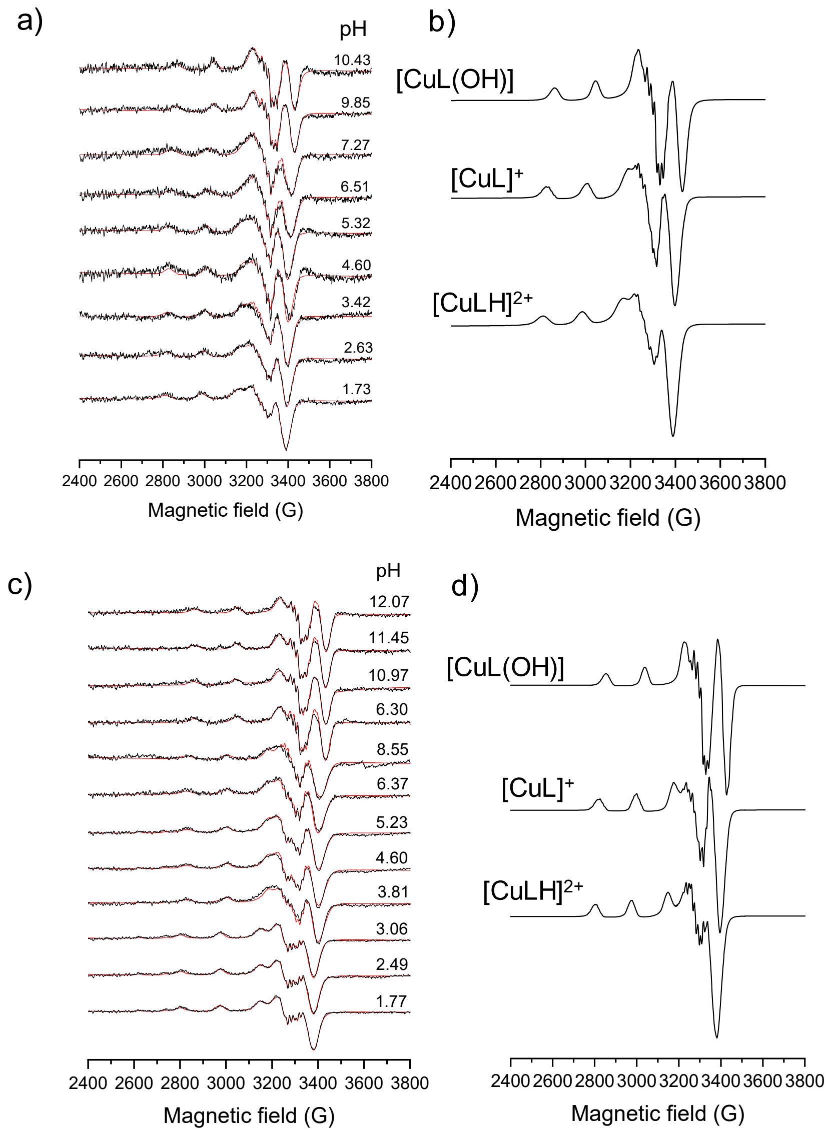

Figure S11. Experimental (black) and simulated (red) frozen solution EPR spectra recorded for the a) copper(II)–COTI-2 and c) copper(II)–COTI-NH_2 systems. Spectra were normalised to the maximum intensity. Calculated EPR spectra obtained for the various complexes of b) COTI-2 and d) COTI-NH_2. $c_{Cu(II)} = c_L$ = 20 µM (COTI-2); $c_{Cu(II)} = c_L$ = 80 µM (COTI-NH_2); 77 K; 30% (v/v) DMSO/H_2O.

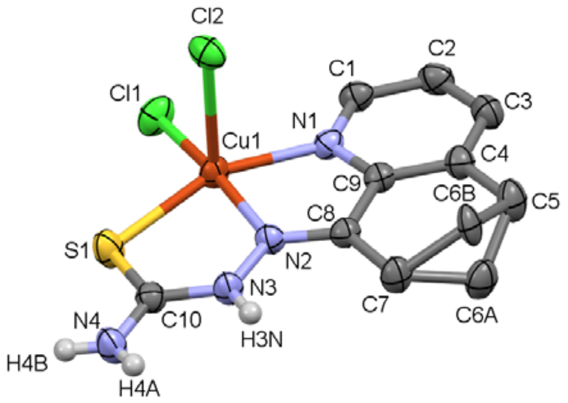

Figure S12. ORTEP view of [Cu(COTI-NH$_2$)Cl$_2$]$\cdot$CH$_3$OH with thermal ellipsoids at 50% probability level. Hydrogen atoms and the methanol solvent are omitted for clarity. The cyclohexene ring of the dihydroquinoline moiety occupies two disordered positions in the crystal lattice (C6A: 73%, C6B: 27%).

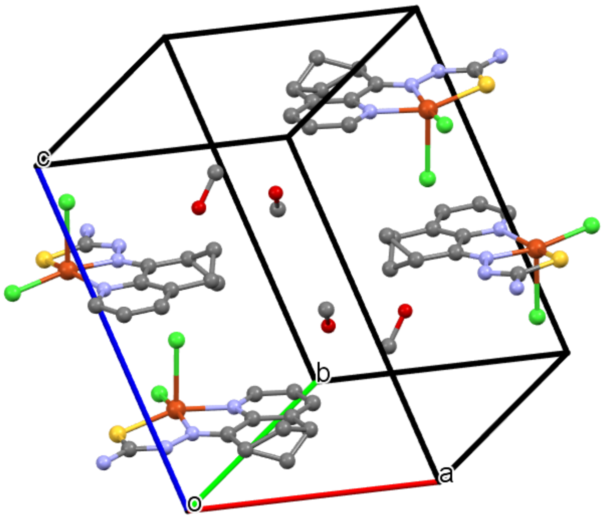

Figure S13. Unit cell containing four molecules of [Cu(COTI-NH$_2$)Cl$_2$]$\cdot$CH$_3$OH.

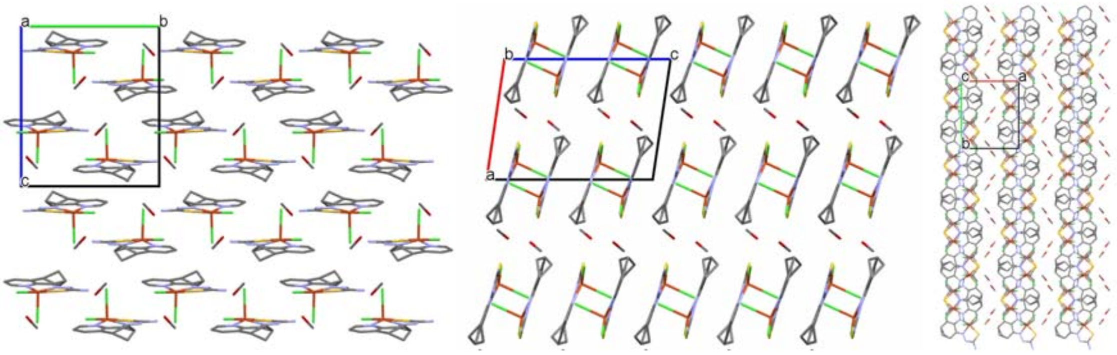

Figure S14. Packing arrangements in the crystal of [Cu(COTI-NH$_2$)Cl$_2$]$\cdot$CH$_3$OH viewed from the crystallographic directions ’a’, ’b’ and ’c’.

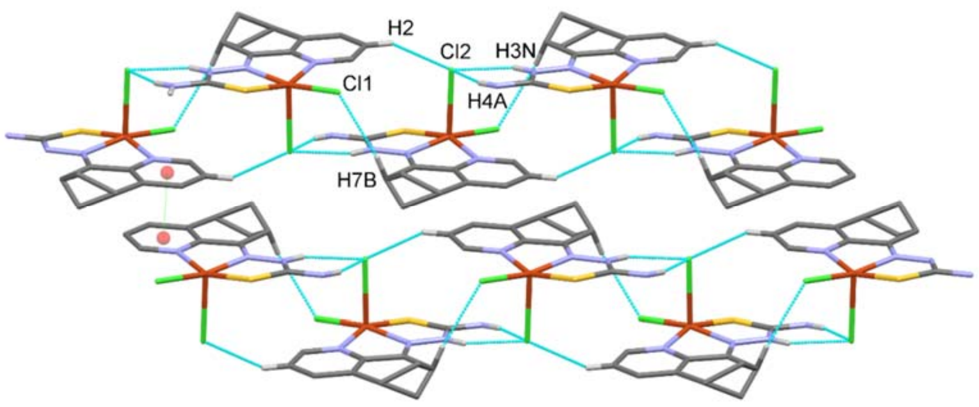

Figure S15. Packing arrangements in the crystal of $[Cu(COTI\text{-}NH_2)Cl_2]^{\cdot}CH_3OH$ showing some selected hydrogen bonds and π…π interactions (3.7732(12) Å).

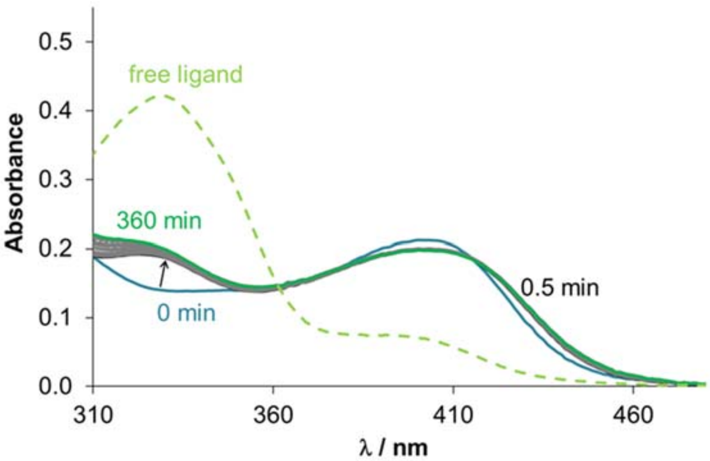

Figure S16. Time-dependent changes of the UV-vis absorption spectra of copper(II)– COTI-NMeCy (1:1) system in the presence of 300 equiv. GSH (solid lines) at pH 7.4 in 5% (v/v) DMSO/H_2O under argon and the spectrum of the free ligand (dashed line). $c_L = c_{Cu(II)}$ = 30 µM; c_{GSH} = 9 mM; T = 25˚C; I = 0.1 M (KCl); ℓ = 1.0 cm.

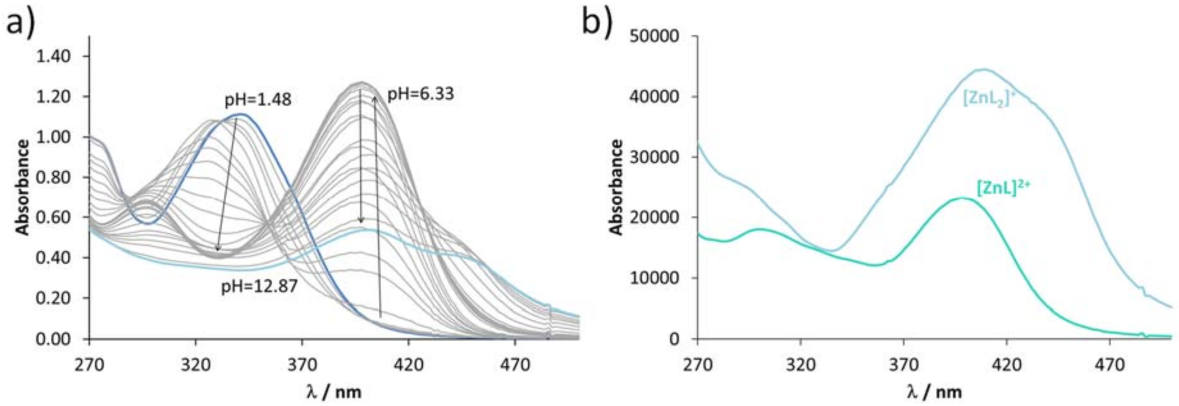

Figure S17. a) UV-vis spectra of the zinc(II)– COTI-NMeCy (1:1) system recorded at various pH values, and b) calculated individual molar absorption spectra of complex species $c_{Zn(II)}$ = c_L = 40 µM; pH = 1.0 – 12.5; T = 25 ˚C; I = 0.10 M (KCl); ℓ = 1.0 cm; 30% (v/v) DMSO/H_2O.

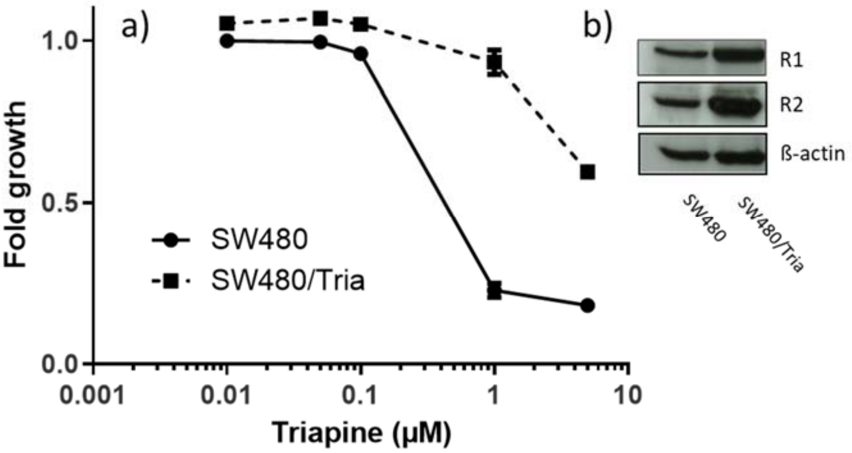

Figure S18. a) Triapine resistance was confirmed in SW480/Tria. Anticancer activity of Triapine was tested by MTT viability assay after 72 h drug incubation in SW480 cells vs. the resistant sublines. Mean ± standard deviation (SD) was derived from triplicates of one representative experiment out of three. b) Protein expression of R1 and R2, was investigated in total protein extracts by Western blotting as previously described [9]. β-actin was used as a loading control.

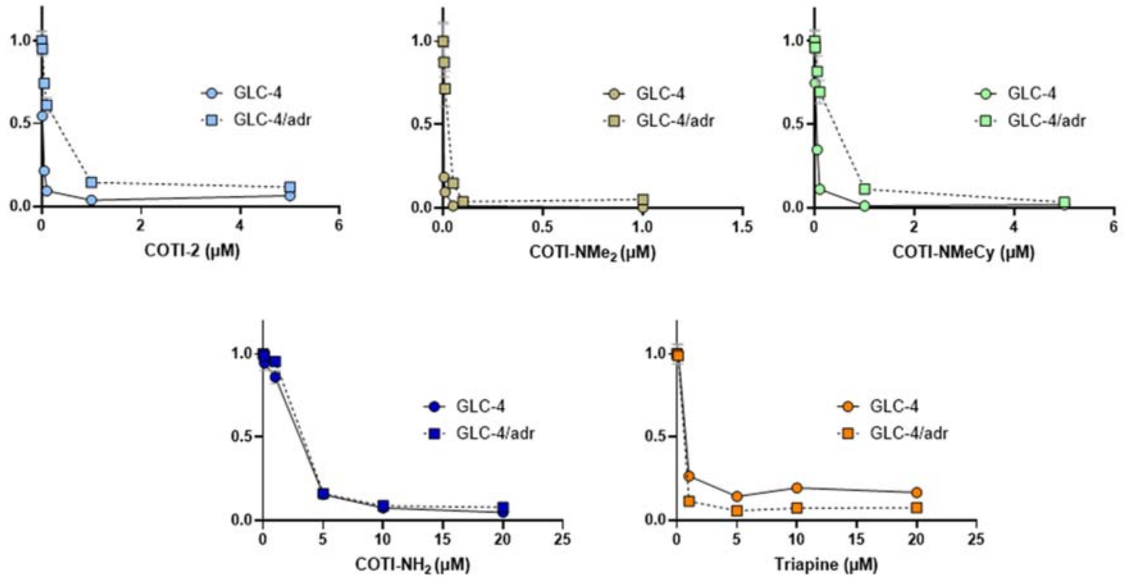

Figure S19. Differences in viability (measured by MTT viability assay) of GLC-4 and ABCC1-overexpressing GLC-4/adr cells treated with COTI-2, COTI-derivatives and Triapine for 72 h. Mean ± standard deviation was derived from triplicates of one representative experiment out of three.

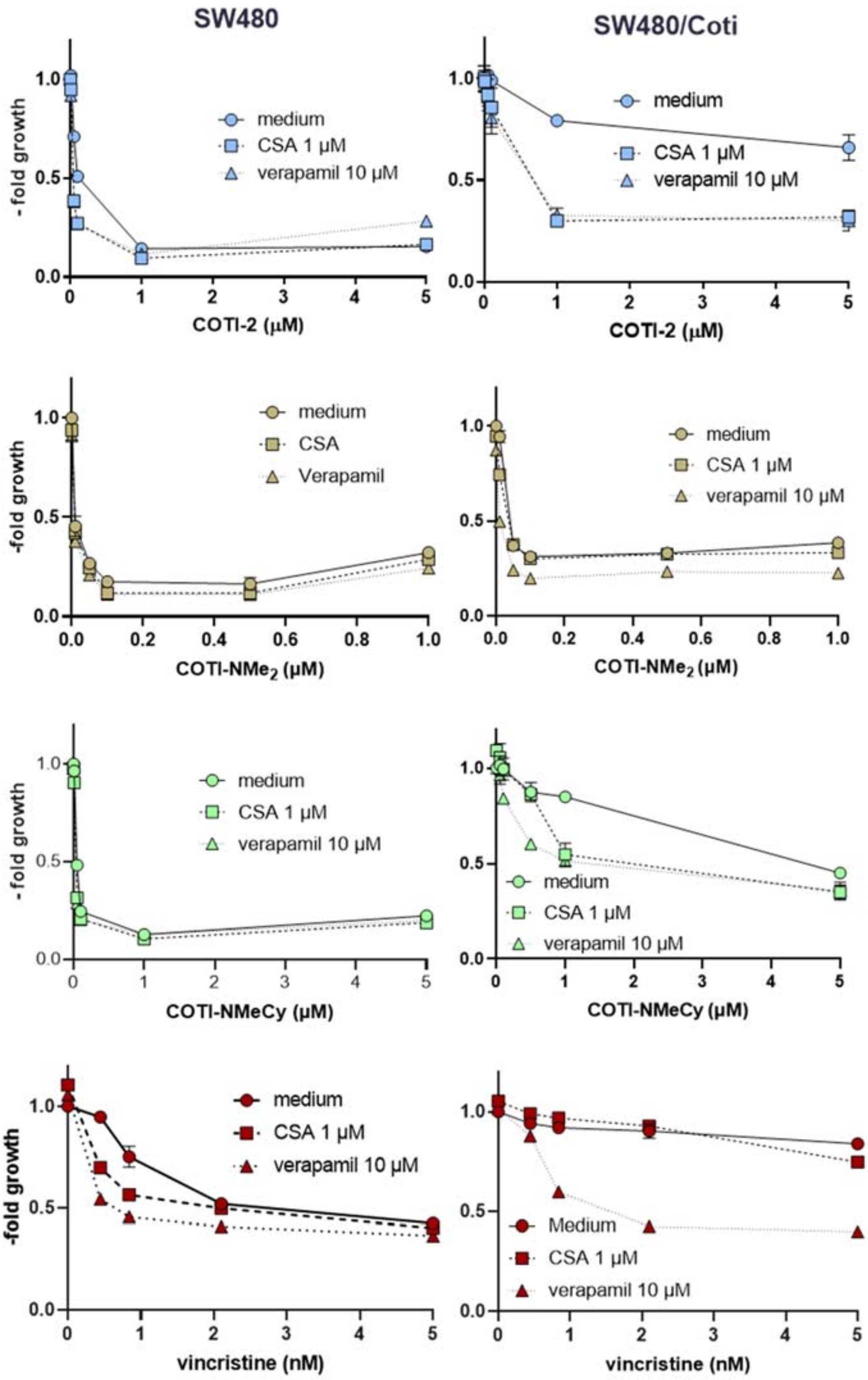

Figure S20. Impact of ABC transporter modulators (1 µM CSA and 10 µM verapamil) on the anticancer activity of vincristine, COTI-2 and its derivatives in the SW480/Coti cells in comparison to the parental cells. Vincristine was used as a positive control of an ABCC1 substrate. Viability was measured by MTT assay after 72 h of combined drug treatment. Mean ± SD was derived from triplicates of one representative experiment out of three.

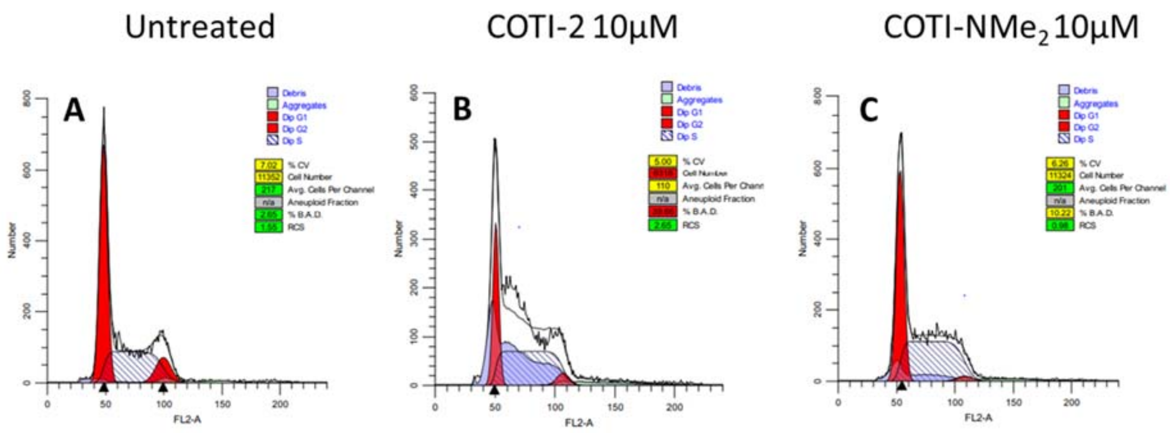

Figure S21. Cell-cycle analysis by PI staining and flow cytometry of SW480 (A;B,C) cells. Cell-cycle analysis by flow cytometry of SW480 untreated cells (A); or treated with 10 µM COTI-2 (B) and 10 µM COTI-NMe_2 (C).

Table S1. Electrochemical data (cathodic and anodic peak potentials *vs.* Ag/AgCl/3 M KCl), peak separation, formal redox potentials *vs.* (NHE) for the copper(II)–ligand (1:1) and iron(III)–ligand (1:2) systems. c_L = 1 mM; pH = 7.40 (10 mM HEPES); T = 25˚C; I = 0.1 M tetrabutylammonium nitrate (TBAN); 90% (v/v) DMSO/H_2O; 0.01 V/s scan rate.

		COTI-2	COTI-NH_2	COTI-NMe_2	COTI-NMeCy	Triapine
**Cu(II)**	**E_a (mV)**	-448	-420	-500	-467	-383
	**E_c (mV)**	-384	-356	-422	-409	-313
	**ΔE (mV)**	64	64	78	58	70
	**E' vs. NHE (V)**	-0.19	-0.17	-0.24	-0.22	-0.13
**Fe(III)**	**E_a (mV)**	-147	-203	-193	-203	-219
	**E_c (mV)**	-88	-127	-130	-144	-160
	**ΔE (mV)**	59	76	63	59	59
	**E' vs. NHE (V)**	+0.11	+0.06	+0.06	+0.05	+0.03

Table S2. Anisotropic EPR spectroscopic parameters obtained for the studied copper(II) complexes of COTI-NH_2 and COTI-2 in 30% (v/v) DMSO/H_2O. {77K}

	g_x	g_y	g_z	A_x (G)	A_y (G)	A_z (G)	a_{Nx} (G)	a_{Ny} (G)	a_{Nz} (G)
**$Cu^{2+}_{(aqua)}$**	2.080	2.080	2.412	8.0	8.0	113.2	–	–	–
**HL=COTI-NH_2**									
**$[CuLH]^{2+}$**	2.051	2.025	2.199	18.9	24.1	168.6	16.4	9.8	10.0
							11.5	16.5	10.4
**$[CuL]^+$**	2.051	2.020	2.180	20.8	29.2	174.0	14.2	7.9	11.9
							8.6	16.8	11.6
**[CuL(OH)]**	2.040	2.025	2.147	24.7	13.2	181.6	16.2	10.7	7.7
							14.5	16.8	10.8
**HL=COTI-2**									
**$[CuLH]^{2+}$**	2.050	2.029	2.188	17.8	38.8	170.6	15.6	12.1	12.0
							9.9	16.2	14.1
**$[CuL]^+$**	2.053	2.022	2.171	18.6	24.8	174.7	13.2	7.7	12.0
							7.2	18.5	12.9
**[CuL(OH)]**	2.040	2.025	2.145	26.9	16.7	179.2	16.2	10.7	7.7
							14.5	16.8	10.8

[a] The experimental error were ±0.001 for *g*, ± 1 G for *A* and a_N.

Table S3. Isotropic EPR parameters calculated for the copper(II) complexes of COTI-NH_2 and COTI-2 from the anisotropic data as an average of the *g* and *A* values (Table 5) and experimentally determined data for the copper(II)-complexes of Triapine [10].

	g_0	A_0 (G)		g_0	A_0 (G)		g_0	A_0 (G)
**HL = COTI-NH_2**			**HL = COTI-2**			**HL = Triapine**		
**$[CuLH]^{2+}$**	2.092	70.5	**$[CuLH]^{2+}$**	2.089	75.7	**$[CuLH]^{2+}$**	2.107	73.4
**$[CuL]^{+}$**	2.084	74.7	**$[CuL]^{+}$**	2.082	72.7	**$[CuL]^{+}$**	2.096	72.6
**[CuL(OH)]**	2.071	73.2	**[CuL(OH)]**	2.070	74.3	**[CuL(OH)]**	2.086	70.7

Table S4. Selected bond lengths (Å) and angles (°) for the complexes in crystal $[Cu(COTI\text{-}NH_2)Cl_2]\cdot CH_3OH$.

Bond	Distance (Å)
Cu1-N1	2.043(2)
Cu1-N2	1.983(2)
Cu1-S1	2.2916(6)
Cu1-Cl1	2.2476(6)
Cu1-Cl2	2.6525(7)
**Bond**	**Angle (°)**
N2-Cu1-N1	79.30(7)
N1-Cu1-Cl1	99.18(5)
N1-Cu1-S1	158.02(5)
N2-Cu1-Cl2	92.66(5)
Cl1-Cu1-Cl2	98.90(2)
S1-Cu1-Cl2	104.79(2)
N2-Cu1-Cl1	168.35(6)

Table S5. Hydrogen-bond geometry of in crystal $[Cu(COTI\text{-}NH_2)Cl_2]\cdot CH_3OH$.

**D-H…A**	**D-H** (Å)	**H…A** (Å)	**D…A** (Å)	**D-H…A** (°)	**symmetry codes**
O1-H1A…Cl1	0.82	2.39	3.208(2)	176	1+x,y,z
N3-H3N…Cl2	0.86	2.32	3.1145(18)	154	-x,-1/2+y,1/2-z
N4-H4A…Cl2	0.79	2.49	3.214(2)	154	-x,-1/2+y,1/2-z
N4-H4B…O1	0.80	2.01	2.800(3)	171	-x,-1/2+y,1/2-z
C2-H2…Cl2	0.93	2.78	3.465(2)	132	-x,1/2+y,1/2-z
C7-H7B…Cl1	0.97	2.77	3.695(2)	159	-x,-1/2+y,1/2-z

Table S6. Crystal data and structure refinement for $[Cu(COTI\text{-}NH_2)Cl_2]{\cdot}CH_3OH$

	$[Cu(COTI\text{-}NH_2)Cl_2]{\cdot}CH_3OH$
Color/Shape	Blue/Block
Empirical formula	$C_{11}H_{16}Cl_2CuN_4OS$
Moiety formula	$C_{10}H_{12}Cl_2CuN_4S$, CH_3OH
Formula weight	386.78
Temperature (K)	295(2)
Radiation and wavelength	Mo-Kα, λ =0.71073 Å
Crystal system, Space group	monoclinic, $P\ 2_1/c$
Unit cell dimensions	
a (Å)	9.9721(3)
b (Å)	11.6151(4)
c (Å)	13.5737(5)
α (°)	90
β(°)	98.449(7)
γ (°)	90
Volume ($Å^3$)	1555.14(10)
Z/Z'	4/1
Density (calculated) (Mg/m^3)	1.652
Absorption coefficient, μ (mm^{-1})	1.882
F(000)	788
Crystal size (mm)	0.50 × 0.20 × 0.15
Absorption correction	Numerical
Max. and min. transmission	0.889, 0.959
Θ–range for data collection (°)	3.034 ≤ Θ ≤ 27.469°
Reflections collected	46670
Completeness to 2Θ	0.999
Independent reflections, (R_{int})	3546, 0.0352
Reflections $I>2\sigma(I)$	3257
Refinement method	full-matrix least-squares on F^2
Data / restraints / parameters	3546 / 0 /193
Goodness-of-fit on F^2	1.195
Final *R* indices [$I>2\sigma(I)$], R_1, wR_2	0.0353, 0.0695
R indices (all data), R_1, wR_2	0.0408, 0.0712
Max. and mean shift/esd	0.000; 0.000
Largest diff. peak and hole (e.$Å^{-3}$)	0.379; -0.203

Supplementary References:

1. Rockenbauer, A.; Korecz, L. Automatic computer simulations of ESR spectra. Applied Magnetic Resonance **1996**, 10, 29-43.

2. Higashi, T. Numerical Absorption Correction 2002.

3. CrystalClear, S.M. 1.4. 0. *Rigaku/MSC Inc* **2008**.

4. Sheldrick, G.M. ShelXL2013. *University of Göttingen, Germany* **2013**.

5. Farrugia, L.J. WinGX and ORTEP for Windows: An Update. *Journal of Applied Crystallography* **2012**, *45*, 849–854.

6. Spek, A.L.J. Single-Crystal Structure Validation with the Program PLATON. *Journal of applied crystallography* **2003**, *36*, 7–13.

7. Macrae, C.F.; Edgington, P.R.; McCabe, P.; Pidcock, E.; Shields, G.P.; Taylor, R.; Towler, M.; Streek, J.V.D. Mercury: Visualization and Analysis of Crystal Structures. *Journal of Applied Crystallography* **2006**, *39*, 453–457.

8. Allen, F.H.; Johnson, O.; Shields, G.P.; Smith, B.R.; Towler, M. CIF Applications. XV. EnCIFer: A Program for Viewing, Editing and Visualizing CIFs. *Journal of applied crystallography* **2004**, *37*, 335–338.

9. Heffeter P, Popovic-Bijelic A, Saiko P, Dornetshuber R, Jungwirth U, Voevodskaya N, Biglino D, Jakupec MA, Elbling L, Micksche M, Szekeres T, Keppler BK, Gräslund A, Berger W. Ribonucleotide reductase as one important target of [Tris(1,10-phenanthroline)lanthanum(III)] trithiocyanate (KP772). *Curr Cancer Drug Targets*. **2009**, 9(5), 595-607.

10. Enyedy, É.A.; Nagy, N. v.; Zsigó, É.; Kowol, C.R.; Arion, V.B.; Keppler, B.K.; Kiss, T. Comparative Solution Equilibrium Study of the Interactions of Copper(II), Iron(II) and Zinc(II) with Triapine (3-Aminopyridine-2-Carbaldehyde Thiosemicarbazone) and Related Ligands. *European Journal of Inorganic Chemistry* **2010**, 1717–1728.
